# Interruption of the long non-coding RNA HOTAIR signaling axis ameliorates chemotherapy-induced cachexia in bladder cancer

**DOI:** 10.1186/s12929-022-00887-y

**Published:** 2022-12-06

**Authors:** Che-Yuan Hu, Bing-Hua Su, Ya-Che Lee, Chung-Teng Wang, Mei-Lin Yang, Wan-Ting Shen, Jing-Ting Fu, Shih-Yao Chen, Wei-Yun Huang, Chien-Hui Ou, Yuh-Shyan Tsai, Feng-Chih Kuo, Ai-Li Shiau, Gia-Shing Shieh, Chao-Liang Wu

**Affiliations:** 1 Institute of Clinical Medicine, College of Medicine, National Cheng Kung University, Tainan, Taiwan; 2 Department of Urology, National Cheng Kung University Hospital, College of Medicine, National Cheng Kung University, 138, Sheng Li Road, Tainan, 704302 Taiwan; 3 School of Respiratory Therapy, College of Medicine, Taipei Medical University, Taipei, Taiwan; 4 Department of Urology, Ditmanson Medical Foundation Chia-Yi Christian Hospital, Chiayi, Taiwan; 5 Department of Microbiology and Immunology, College of Medicine, National Cheng Kung University, 1 University Road, Tainan, 701401 Taiwan; 6 Ditmanson Medical Foundation Chia-Yi Christian Hospital, Chiayi, Taiwan; 7 Department of Biochemistry and Molecular Biology, College of Medicine, National Cheng Kung University, 1 University Road, Tainan, 701401 Taiwan; 8 Department of Nursing, College of Nursing, Chung Hwa University of Medical Technology, Tainan, Taiwan; 9 Division of Endocrinology and Metabolism, Department of Internal Medicine, Tri-Service General Hospital, National Defense Medical Center, Taipei, Taiwan; 10 Department of Urology, Tainan Hospital, Ministry of Health and Welfare, Executive Yuan, Tainan, Taiwan

**Keywords:** HOTAIR, Cachexia, Cisplatin, Bladder cancer, Prothymosin α, EGFR, Chemotherapy

## Abstract

**Background:**

Cisplatin-based chemotherapy is the first line of treatment for bladder cancer. However, cisplatin induces muscle wasting associated with NF-κB and cancer cachexia. HOTAIR, an oncogenic long non-coding RNA (lncRNA), promotes cancer progression in different cancers. Crosstalk between HOTAIR and NF-κB is documented. Prothymosin α (ProT) plays important roles in cancer progression and inflammation. However, the potential link between HOTAIR, ProT, and cisplatin-induced cancer cachexia remains unexplored. Here, we investigated the contribution of HOTAIR in cisplatin-induced cancer cachexia and dissected the potential signaling cascade involving the epidermal growth factor receptor (EGFR), ProT, NF-κB, and HOTAIR.

**Materials and methods:**

Expression of ProT and HOTAIR transcripts and their correlations in tumor tissues of bladder cancer patients and bladder cancer cell lines were determined by RT-qPCR. Next, levels of phospho-EGFR, EGFR, phospho-NF-κB, and NF-κB were examined by immunoblot analysis in human bladder cancer cells treated with cisplatin. Expression of HOTAIR in cisplatin-treated cells was also assessed by RT-qPCR. Pharmacological inhibitors and overexpression and knockdown approaches were exploited to decipher the signaling pathway. The murine C2C12 myoblasts were used as an in vitro muscle atrophy model. The syngeneic murine MBT-2 bladder tumor was used to investigate the role of mouse Hotair in cisplatin-induced cancer cachexia.

**Results:**

Expression of ProT and HOTAIR was higher in bladder tumors than in normal adjacent tissues. There were positive correlations between ProT and HOTAIR expression in clinical bladder tumors and bladder cancer cell lines. Cisplatin treatment increased EGFR and NF-κB activation and upregulated ProT and HOTAIR expression in bladder cancer cells. ProT overexpression increased, whereas ProT knockdown decreased, HOTAIR expression. Notably, cisplatin-induced HOTAIR upregulation was abrogated by EGFR inhibitors or ProT knockdown. ProT-induced HOTAIR overexpression was diminished by NF-κB inhibitors. HOTAIR overexpression enhanced, whereas its knockdown reduced, cell proliferation, cachexia-associated pro-inflammatory cytokine expression, and muscle atrophy. Cachexia-associated symptoms were ameliorated in mice bearing Hotair-knockdown bladder tumors undergoing cisplatin treatment.

**Conclusions:**

We demonstrate for the first time a critical role for HOTAIR and identify the involvement of the EGFR-ProT-NF-κB-HOTAIR signaling axis in cisplatin-induced cachexia in bladder cancer and likely other cancers. Our findings also provide therapeutic targets for this disease.

**Supplementary Information:**

The online version contains supplementary material available at 10.1186/s12929-022-00887-y.

## Background

Cancer cachexia defined as a complex metabolic disorder associated with cancer progression is characterized by body weight loss and skeletal muscle wasting with or without fat mass loss. Remarkably, cachexia rather than tumor burden is responsible for approximately one-third of cancer-related deaths [1]. Cachexia negatively affects patients’ quality of life and physical function and is associated with poor prognosis and survival. While there has been great progress in understanding the pathophysiology of cachexia, no specific treatments or interventions are currently available. Cancer cachexia is regulated by multiple signaling pathways [2]. A correlation between cachexia and systemic chronic inflammation is well documented in cancer patients [3, 4]. Cancer cachexia is accompanied by increased releases of pro-inflammatory cytokines. Among these are interleukin (IL)-6, tumor-necrosis factor α (TNF-α), and IL1-β [5]. Accumulating preclinical evidence has indicated that systemic inflammation is both sufficient and required for cancer cachexia [2]. Direct and indirect effects of systemic inflammation can result in muscle wasting and cachexia. NF-κB is activated by numerous inflammatory stimuli, DNA damage response, and oxidative stress, resulting in inducing expression of various inflammatory mediators [6]. NF-κB and its downstream pathways play a pivotal role in the pathogenesis of muscle atrophy by enhancing inflammation, increasing muscle-specific E3 ubiquitin ligases, such as muscle RING finger-1 (MuRF1) and atrogin-1/MAFbx, and inhibiting myogenesis [7].

A systematic literature review has indicated that sarcopenia occurs in about 25–69% of patients with muscle-invasive bladder cancer and about 52–76% of patients with inoperable locoregionally advanced and/or metastatic disease [8]. Chemotherapy is the mainstream treatment for these patients; however, it may hasten the progression of cachexia [9]. Bladder cancer is the most prevalent malignancy of the urinary tract worldwide. Moreover, 25% of bladder cancers are muscle-invasive or metastatic that may be treated with chemotherapeutic agents as neoadjuvant, adjuvant, or systemic chemotherapy. Cis-diammine-dichloroplatinum (II) (also known as cisplatin or CDDP) is one of the most widely used chemotherapeutic agents for bladder cancer. However, the most relevant severe side of cisplatin chemotherapy is cachexia, primarily due to muscle-wasting-associated body weight loss [10–12]. Epidermal growth factor receptor (EGFR), a member of the ErbB family, plays important roles in mediating both cell proliferative and survival signals. EGFR, which is frequently mutated and/or overexpressed in different cancers, has prognostic significance and is the target of multiple cancer therapies [13]. EGFR positivity is also associated with bladder cancer progression [14]. Treatment of human glioma cells and breast cancer cells with cisplatin induces activation of the EGFR and promotes cell survival [15]. Furthermore, the increased EGFR phosphorylation following cisplatin treatment is in a ligand-independent manner, but requires EGFR kinase activity [15]. Several extracellular stimuli, such as agonists of the G-protein-coupled receptors (GPCRs), can transactivate the EGFR [16]. Protease-activated receptor 2 (PAR-2), a member of a family of GPCRs, is frequently overexpressed in various human cancers, and that PAR-2-induced cisplatin resistance is dependent on EGFR transactivation in cervical cancer [17]. Cisplatin also induces nuclear import of the EGFR, which results in enhanced DNA repair and cell survival after DNA damage [18]. Understanding the molecular signaling pathways involved in cisplatin-induced bladder cancer cachexia may not only identify potential therapeutic targets for cachexia, but also advance therapeutic interventions to cancer treatment.

HOTAIR (HOX transcript antisense intergenic RNA) is a long non-coding RNA (lncRNA) transcribed from the antisense strand of the *HOXC* gene cluster on chromosome 12 and acts to silence *HOXD* genes required for limb development [19]. HOTAIR interacts with various chromatin-modifying complexes and thus acts as a molecular scaffold to change the chromatin state, thereby inducing transcriptional repression of target genes [20, 21]. HOTAIR is overexpressed in a wide range of human cancers and correlated with tumor progression and poor prognosis [22, 23]. It regulates various genes involved in tumorigenesis, tumor invasion and metastasis, epithelial-mesenchymal transition (EMT), chemoresistance, and radioresistance [23]. In addition, HOTAIR also links to inflammation via regulating the NF-κB signaling pathway. Putative NF-κB binding sites within the HOTAIR promoter region was identified [24]. In ovarian cancer, NF-κB transcriptionally upregulates HOTAIR expression during cisplatin-induced DNA damage, and HOTAIR facilitates degradation of the NF-κB inhibitor IκBα and thus enhances NF-κB activation, thereby creating a feed-forward regulatory circuit between NF-κB and HOTAIR [25]. This feed-forward NF-κB/HOTAIR signaling axis was also reported in lipopolysaccharide-induced cytokine expression and inflammatory responses in macrophages [26]. Emerging evidence has shown that lncRNAs can modulate sarcopenia-related signaling pathways [27]. The involvement of HOTAIR in cancer progression and NF-κB signaling suggests that HOTAIR may also be an important player in cancer cachexia. To date, there have been no investigations into the role of HOTAIR and the signaling pathway involved in cancer-induced or chemotherapy-induced cachexia.

Prothymosin α (ProT), an evolutionarily conserved acidic small protein, exerts both intracellular and extracellular functions in health and disease [28]. Intracellular roles of ProT involve gene regulation, cell proliferation, anti-apoptosis, and more. We reported that ProT participates in cell proliferation by shortening the duration of the G1 phase of the cell cycle [29]. We demonstrated that ProT can increase NF-κB acetylation by dissociating histone deacetylases (HDAC) from NF-κB and by facilitating the recruitment of the histone acetyltransferase (HAT) p300 to NF-κB [30]. Moreover, ProT enhances the stability and transcriptional activity of NF-κB, thereby upregulating the expression of NF-κB-dependent genes [30]. We also reported that ProT participates in the induction of insulin resistance through the Toll-like receptor 4 (TLR4)-NF-κB-dependent signaling pathway [31]. STAT3 signaling is a major intrinsic pathway for cancer inflammation. We have demonstrated that ProT enhances STAT3 phosphorylation, which results in STAT3 activation, and promotes STAT3 acetylation required for its stabilization and transcriptional activity [32]. In addition to its intracellular functions, ProT also acts extracellularly to modulate immune responses similarly to molecules named as “alarmins” [33]. Our prior work indicates that ProT can be used as an adjuvant to enhance vaccine efficacy [34, 35]. Extracellular ProT binds to TLR4/myeloid differentiation 2 (MD-2) and activates downstream signaling through the TRIF (TIR-domain-containing adapter-inducing interferon-β)-dependent pathway [36, 37]. We also showed that ProT is highly expressed in tumor tissues and urine of bladder cancer patients and may be served as a tumor marker [38, 39]. We have previously reported that transgenic mice overexpressing ProT, which induces polycystic kidney disease, express elevated EGFR transcripts in the kidney [40]. Although ProT plays important roles in cancer progression and inflammation, its role in cancer cachexia has yet to be explored.

In the present study, we hypothesized that treatment of bladder cancer cells with cisplatin would induce upregulation of HOTAIR, resulting in increases in pro-inflammatory cytokine expression and subsequent cancer cachexia, and that this process is mediated, at least in part, through the EGFR-ProT-NF-κB-HOTAIR signaling axis. We further hypothesized that interruption of this signaling axis with EGFR or NF-κB inhibitors of silencing of ProT or HOTAIR would ameliorate cisplatin-induced bladder cancer cachexia. Our results obtained from cell culture, clinical samples, and animal models elucidate for the first time the pathological role for HOTAIR in cisplatin-induced bladder cancer cachexia. Furthermore, we identify a novel molecular mechanism involving the EGFR-ProT-NF-κB-HOTAIR signaling axis in bladder cancer cachexia induced by cisplatin chemotherapy. Our findings also suggest that the components of this signaling axis may be novel therapeutic targets in cisplatin-induced cachexia in bladder cancer and likely other cancers.

## Materials and methods

### Clinical samples

Patients who underwent transurethral resection of bladder tumor or radical cystectomy between September 2011 and December 2018 at National Cheng Kung University (NCKU) Hospital were enrolled in this study. The inclusion criteria included at least 18 years of age and non-use of neoadjuvant chemotherapy or preoperative radiotherapy during cancer treatment. The exclusion criteria included a previous history of diabetes mellitus, metabolic disorder, or cachexia. Randomization and blinding were not applicable in this study. The ethics committee of NCKU Hospital approved the study, and informed consent was obtained from each patient (IRB number: A-ER-106-451).

### Cell lines, mice, and chemical reagents

Six human bladder cancer, one renal pelvis transitional cell carcinoma (BFTC909), and one prostate carcinoma (PC3) cell lines were used in this study. J82 and TCCSUP cancer cell lines were originally obtained from American Type Culture Collection (ATCC, Manassas, VA, USA). Human BFTC905, BFTC909, T24, and HT1197 cancer cell lines, as well as a normal human uroepithelial cell line (SV-HUC-1) were purchased from Bioresource Collection and Research Center (Hsinchu, Taiwan). Human TSGH-8301 and murine MBT-2 bladder cancer cell lines were originally obtained from M.Y. Yeh (Tri-Service General Hospital, Taipei) and C.R. Yang (Taichung Veterans General Hospital, Taichung), respectively. Mouse C2C12 myoblasts were a gift from Y.S. Shan (Institute of Clinical Medicine, NCKU). Human 293 T embryonic kidney cells were obtained from the National RNAi Core (Academia Sinica, Taiwan). All cells, unless stated otherwise, were cultured in Dulbecco’s Modified Eagle’s Medium (DMEM) supplemented with 10% cosmic calf serum (HyClone, Logan, UT, USA) or fetal bovine serum (FBS, HyClone), 2 mmol/L L-glutamine, and 50 μg/ml gentamicin at 37 °C in an atmosphere of 5% CO_2_. Murine C2C12 myoblasts were maintained in DMEM supplemented with 10% FBS and not allowed to reach confluence to avoid differentiation. For animal studies, 6–8-week-old C3H/HeN and C57BL/6 mice were purchased from NCKU Laboratory Animal Center or National Laboratory Animal Center (Taipei, Taiwan). Cisplatin (KEMOPLAT) and 4-methyl-*N*1-(3-phenyl-propyl)-benzene-1,2-diamine (JSH-23) were obtained from Fresenius Kabi Oncology Limited (Solan, India) and Santa Cruz Biotechnology (Santa Cruz, CA, USA), respectively. Gefitinib, erlotinib, and 2-[(aminocarbonyl)amino]-5-(4-fluorophenyl)-3-thiophenecarboxamide (TPCA-1) were purchased from Cayman Chemical Company (Ann Arbor, MI, USA).

### Lentiviral vectors and CRISPR interference (CRISPRi)

We have previously constructed the lentiviral vector pSin-EF2-ProT-Pur encoding human ProT [31]. To construct the control vector pSin-EF2-EGFP-Pur that encodes EGFP, the coding region of Oct4 was removed from pSin-EF2-Oct4-Pur (Addgene plasmid 16579) by *Spe*I and *Eco*RI digestion and replaced with the coding sequence of EGFP obtained from pEGFP-C1 (Clontech, Palo Alto, CA, USA) by digestion with *Nhe*I and *Mfe*I. The lentiviral vector encoding human HOTAIR (pEGFP-Lv105-HOTAIR) was previously described [41]. pAll-dCas9-KRAB.pPuro plasmid was provided by the RNAiCore, and the guide RNA (gRNA) sequences were designed as previously described [42]. To generate CRISPRi-containing lentiviral vectors targeting mouse HOTAIR (hereafter Hotair), the designed gRNA sequences for Hotair-F4 5′-GGGGUUUCAGCCGGAAGUGG-3′, mHotair-F6 5′-GUGAUCUGAGUCUCCUUUAAA-3′, and GFP (the control gRNA) 5′-GGGCGAGGAGCUGUUCACCG-3′ were generated from the following guide oligos: F4, 5′-GGGGTTTCAGCCGGAAGTGG-3’ (sense) and 5′- CCACTTCCGGCTGAAACCCC-3′ (anti-sense); F6, 5′-GTGATCTGAGTCTCCTTTAAA-3′ (sense) and 5′- TTTAAAGGAGACTCAGATCAC-3′ (anti-sense), and GFP, 5′-GGGCGAGGAGCTGTTCACCG-3′ (sense) and 5′- CGGTGAACAGCTCCTCGCCC-3′ (anti-sense), respectively. The three annealed guide oligos (F4, F6, and GFP) were individually cloned into the *Bsm*BI sites of pAll-dCas9-KRAB.pPuro to replace a 1.9-kb stuffer sequence. For shRNA-based knockdown of ProT, pLKO.1-puro-based lentiviral vectors including stem-loop cassettes encoding shRNA for human ProT (TRCN0000134223 and TRCN0000138110; designated shProT-17 and shProT-19, respectively) and luciferase (TRCN0000072246; designated shLuc) were used [30].

Various recombinant lentiviruses expressing human HOTAIR, human ProT, GFP, shProT-17, shProT-19, shLuc, CRISPRi (CSi)-Hotair-F4, CSi-Hotair-F6, and CSi-GFP were produced by transient transfection of 293 T cells with the aforementioned lentiviral expression vectors along with the packaging plasmid psPAX2 and the VSV-G expression plasmid pMD2.G as previously described [31, 43].

### Reverse transcription quantitative real-time polymerase chain reaction (RT-qPCR)

Total RNA from cells was extracted using the RNeasy Mini kit (Qiagen, Hilden, Germany). The miRNeasy FFPE kit (Qiagen), which is specifically designed for formalin-fixed, paraffin-embedded materials and contains reagents to reverse formalin cross-linking of RNA, was used to extract total RNA from formalin-fixed, paraffin-embedded tissue specimens. Total RNA was reverse transcribed into cDNA with the High-Capacity cDNA Reverse Transcription kit (Thermo Fisher Scientific, Waltham, MA, USA). The qPCR was performed with TOOLS 2xSYBER qPCR Mix (Biotools, Taiwan) on a StepOnePlus Real-Time PCR system (Thermo Fisher Scientific). Relative mRNA expression was determined using the 2-ΔΔCt Method, with value obtained by subtracting the Ct value of glyceraldehyde-3-phosphate dehydrogenase (GAPDH) mRNA from the Ct value of the target mRNA. The following primers were used: human HOTAIR, 5′-GGTAGAAAAAGCAACCACGAAGC-3′ (sense) and 5′-ACATAAACCTCTGTCTGTGAGTGCC-3′ (anti-sense); mouse Hotair, 5′- CCCATCTTTATGACGAGGCTTGTTAA-3′ (sense) and 5’-GCAGACATATTGTTTATGAGTCCACAGG-3′ (anti-sense); human and mouse ProT, 5′-AAGGAGAAGAAGGAAGTTGTGGA-3′ (sense) and 5’-CTACCTCATTGTCAGCCTCCTG-3′ (anti-sense); human IL-6, 5′-ACTCACCTCTTCAGAACGAATTG-3′ (sense) and 5′-CATCTTTGGAAGGTTCAGGTTG-3′ (anti-sense); mouse IL-6, 5′-AGTTGCCTTCTTGGGACTGA-3′ (sense) and 5’-TCCACGATTTCCCAGAGAAC-3′ (anti-sense); human TNF-α, 5′-CTCACATACTGACCCACGGC-3′ (sense) and 5′-AGGAGAAGAGGCTGAGGAACA-3′ (anti-sense); mouse TNF-α, 5′- CTACTGAACTTCGGGGTGATCG-3′ (sense) and 5′-CAGCCTTGTCCCTTGAAGAGAA-3′ (anti-sense); human IL-1β, 5′-ATTACAGTGGCAATGAGGATGAC-3′ (sense) and 5′-CTGTAGTGGTGGTCGGAGATTC-3′ (anti-sense); mouse IL-1β, 5′-GAAATGCCACCTTTTGACAGTG-3′ (sense) and 5′-CTGGATGCTCTCATCAGGACAG-3′ (anti-sense);. human GAPDH, 5′-ACTTCAACAGCGACACCCACT-3′ (sense) and 5′-GCCAAATTCGTTGTCATACCAG-3′ (anti-sense); as well as mouse GAPDH, 5′-GTTGTCTCCTGCGACTTCAACA-3′ (sense) and 5′-TTGCTGTAGCCGTATTCATTGTC-3′ (anti-sense).

### Immunoblotting, histochemistry, immunohistochemistry, and enzyme-linked immunosorbent assay (ELISA)

Immunoblotting was performed using standard protocols. The primary antibodies used for immunoblotting included anti-PTMA (i.e. ProT; GTX56113, GeneTex, Irvine, CA, USA), anti-EGFR (D38B1) (#4267, Cell Signaling), anti-Phospho-EGFR (Tyr1068) (D7A5) (#3777, Cell Signaling, Danvers, MA, USA), anti-NF-κB p65 (F-6) (sc-8008, Santa Cruz, Dallas, TX, USA), anti-Phospho-NF-κB p65 (Ser536) (93H1) (#3033, Cell Signaling), anti-MuRF-1 (#4305, Cell Signaling), anti-UBR2 (18853-1-AP, Proteintech, Rosemont, IL, USA), anti-atrogin-1 (AP2041, ECM Biosciences, Versailles, KY, USA), and anti-β-actin-peroxidase (A3854, Sigma-Aldrich, St. Louis, MO, USA) antibodies. Secondary antibodies were peroxidase-AffiniPure goat anti-rabbit (111-035-003), or anti-mouse (115-035-003) from Jackson Immunoresearch Laboratories (West Gove, PA, USA). Antibody and antigen complexes were detected using the ECL system (Millipore, Bedford, MA, USA) and visualized with a Biospectrum AC imaging system (UVP, Upland, CA, USA).

Formalin-fixed, paraffin embedded mouse gastrocnemius muscle tissue sections were examined with hematoxylin and eosin (H&E) staining to determine the myofiber cross-sectional area using the ImageJ software (U.S. National Institute of Health) as previously described [44]. For immunohistochemical staining, tissue sections were deparaffinized, rehydrated, and blocked with bovine serum albumin according to standard methods. Subsequently, they were incubated with anti-MuRF-1 rabbit polyclonal antibody (#4305, Cell Signaling) at 4 °C overnight, and then incubated with HRP-conjugated goat anti-rabbit IgG (Jackson) at room temperature for 2 h. The reactivity was visualized with aminoethyl carbazole (AEC, red color, Zymed, South San Francisco, CA, USA) and counterstained with hematoxylin. Mouse serum IL-6 concentrations as well as human and mouse IL-6, TNF-α, and IL-1β levels in the conditioned medium (CM) of J82 and MBT-2 cells and their derivatives were quantified using DuoSet ELISA Kits (R&D, Minneapolis, MN, USA).

### Cell proliferation and drug sensitivity assays

Cell proliferation was determined using the CellTiter 96 AQueous non-reactive cell proliferation assay kit (Promega, Madison, WI, USA) according to manufacturer’s instructions. Briefly, J82 or MBT-2 cells and their derivatives were plated at 4 × 10^3^ cells/well in 96-well plates and incubated for 24, 48, and 72 h. The MTS/PMS reagent (20 μl) was added to each well and incubated for a further 3.5 h. The plates were read at an absorbance of 490 nm that stands for cell growth using an ELISA reader. To assess cisplatin sensitivity, MBT-2 cells and their derivatives (5 × 10^3^ cells/well) that had been cultured in 96-well plates overnight were refed with fresh medium containing various concentrations of cisplatin. After 24 h, cell viability was assessed with the colorimetric WST-8 assay. The 50% inhibitory concentration (IC_50_) values were determined as the drug concentration at 50% inhibition of cell growth.

### Treatment of myotubes with cancer cell-derived CM and measurement of myotube diameter

The CM was collected from cancer cells for treating myotubes as previously described with minor modification [45]. Cancer cells and their derivatives were cultured in DMEM supplemented with 10% FBS at a density of 1 × 10^5^ cells/well in 12-well plates overnight, and then the medium was changed to 1.5 ml of fresh culture medium with or without cisplatin (2 μg/ml). After further incubation for 48 h, the CM was collected and centrifuged to remove cell debris, and the supernatant was stored at − 20 °C until use. C2C12 myoblasts were cultured in DMEM supplemented with 10% FBS at a density of 1.2 × 10^5^ cells/well in 12-well plates. When cells reached 70%-80% confluency, the medium was then switched to the differentiation medium containing DMEM with 2% horse serum (HyClone), and myoblasts were allowed to differentiate into myotubes for 4 days. The CM was collected from cancer cells and diluted 1:1 with fresh DMEM containing 2% horse serum for treating myotubes for 48 h. Additionally, myotubes treated with DMEM containing 100 ng/ml of recombinant proteins (R&D), including mouse TNF-α, human TNF-α, and human IL-6, were used as positive controls for assessing myotube atrophy. The serum content was standardized, so that the CM from cancer cells, untreated control medium, and positive control cytokines had the same final serum concentration and composition. The diameter of myotubes was measured on images using the ImageJ software as previously described with minor modification [46]. Briefly, a total of 105 myotubes (15 myotubes/filed) within each section were measured in randomly selected seven different view fields to calculate the average diameters.

### Animal studies

The experimental protocol adhered to the rules of the Animal Protection Act of Taiwan and was approved by the Institutional Animal Care and Use Committee (IACUC) of NCKU (IACUC approval numbers: 106170 and 107,231). To assess whether knockdown of Hotair could alleviate cisplatin-induced cachexia in the syngeneic MBT-2 tumor model, mice bearing bladder tumors with or without Hotair knockdown were treated with cisplatin. Groups of male C3H/HeN mice were subcutaneously inoculated into the dorsal flank with 3 × 10^6^ of MBT-2, HOTAIR-knockdown MBT-2 (MBT-2/CSi-Hotair-F4 or -F6), or GFP-knockdown MBT-2 (MBT-2/CSi-GFP) cells at day 0. Another group of mice that received vehicle treatment served as the healthy control. Except for healthy control mice, all mice were treated intraperitoneally with cisplatin (5 mg/kg/day) at days 17, 19, 21, 23, 25, and 27. The mice were sacrificed, and tumors were excised and weighed at day 30.

To further examine the effects of cisplatin and/or erlotinib on the tumor growth and cachexia in mice bearing bladder tumors, C3H/HeN mice were inoculated with MBT-2 cells following the same condition as the aforementioned experiment. Subsequently, the mice were intraperitoneally treated with erlotinib (10 mg/kg/day) daily from day 15 to day 21 in combination with cisplatin (5 mg/kg/day) at days 15, 17, 19, and 21 and sacrificed at day 23. We also used another type of bladder tumor model to examine the impact of cisplatin on body weight and tumor growth. C57BL/6 mice were subcutaneously inoculated with 3 × 10^6^ of MB49/shLuc cells that express shLuc at day 0 with or without cisplatin treatment (5 mg/kg/day) at days 15, 17, 19, 21, and 23. The observation period ended at day 25. In all the animal experiments, body weight and palpable tumors of the mice were measured every two or three days. Palpable tumor was measured in two perpendicular axes with a tissue caliper, and tumor volumes were calculated as: (length of tumor) × (width of tumor)^2^ × 0.45. The calf circumference, which has been proposed as a good predictor of muscle mass, was measured with a tissue caliper and calculated using the following geometric formula: 2π(√[a^2^ + b^2^/2]), where *a* and *b* are the laterolateral and anteroposterior diameters, respectively, as described previously [47].

### Statistical analysis

Data are expressed as mean ± standard deviation (SD). Statistical differences were compared by paired *t*-test between paired samples and by one-way analysis of variance (ANOVA) with Bonferroni post hoc test among three or more groups. Correlations were analyzed using Pearson’s correlation coefficient (*r*). Differences in body weight and tumor volume between groups were compared by repeated-measures two-way ANOVA. Any *p* value of < 0.05 was regarded statistically significant. Statistical tests were performed using GraphPad Prism (version 8.0, GraphPad software, San Diego, CA, USA).

## Results

### Expression of ProT and HOTAIR is elevated in bladder tumors and shows a positive correlation

**Fig. 1:**
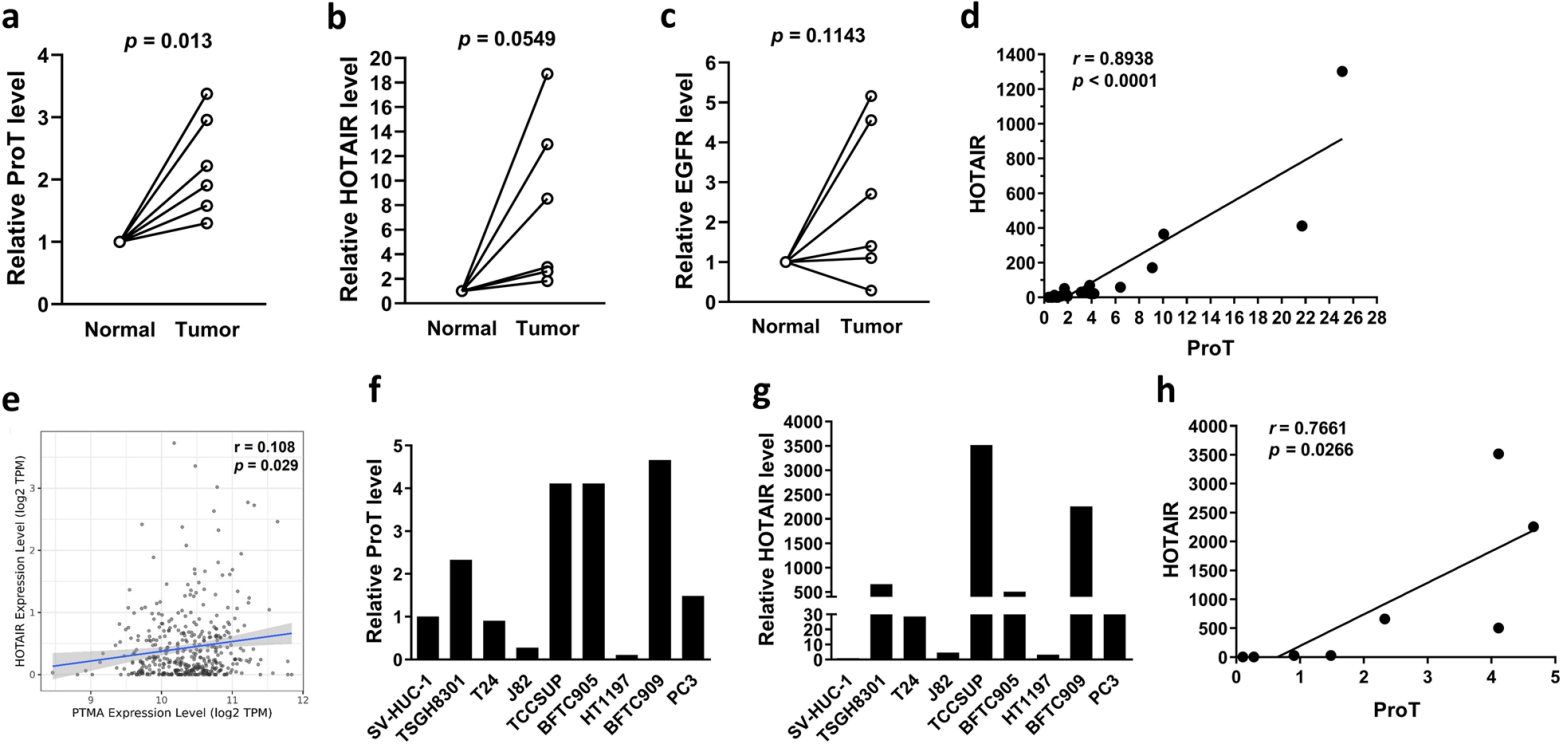
Expression of ProT and HOTAIR is elevated in bladder tumors and shows a positive correlation. **a-c** Quantification of ProT (**a**), HOTAIR (**b**), and EGFR (**c**) transcripts in six pairs of bladder tumor tissues and corresponding adjacent normal tissues by RT-qPCR. **d** Pearson correlation analysis of ProT and HOTAIR transcripts in tumor tissues from 19 patients with bladder cancer showing the best-fit linear trend line. **e** A positive correlation between the expression levels of ProT (PTMA) and HOTAIR transcripts analyzed from the TCGA bladder cancer cohort (n = 408). **f–h** Quantification of ProT (**f**) and HOTAIR (**g**) transcripts in different urothelial carcinoma and normal uroepithelial (SV-HUC-1) cells by RT-qPCR and a positive correlation between the expression levels of ProT and HOTAIR transcripts (**h**)

To study the contribution of HOTAIR and its potential upstream EGFR-ProT axis to cisplatin-induced bladder cancer cachexia, we first asked whether HOTAIR, ProT, and EGFR were overexpressed in clinical bladder tumor tissues. We examined their expression levels in six pairs of bladder tumor tissues and corresponding adjacent normal tissues by RT-qPCR. Expression of ProT (Fig. 1a) and HOTAIR (Fig. 1b) was significantly higher in tumor tissues than their normal tissue counterparts. However, no significant changes in the expression of EGFR were observed in bladder tumors compared to their normal tissue counterparts (Fig. 1c). We further confirmed their association in tumor tissues from 19 patients with bladder cancer. Figure 1d shows that ProT expression was highly positively correlated with HOTAIR expression (*r* = 0.8939, *p* < 0.0001). In addition, we analyzed clinical data from The Cancer Genome Atlas (TCGA) bladder cancer cohort (n = 408) [48]. We examined the intercorrelation between ProT (i.e. PTMA gene), HOTAIR, and EGFR transcripts in bladder tumor samples with the use of the correlation module from the Tumor Immune Estimation Resource (TIMER, https://cistrome.shinyapps.io/timer) web server [49]. Figure 1e shows that there was a positive correlation between the expression levels of ProT and HOTAIR (*r* = 0.108, *p* = 0.029). Nevertheless, expression levels between EGFR and HOTAIR (Additional file 1: Fig. S1a) and between EGFR and ProT (Additional file 1: Fig. S1b) showed no significant correlations. We also examined the expression levels of ProT and HOTAIR in different urothelial carcinoma cell lines, including six bladder cancer, one prostate cancer (PC3), one renal cancer (BFTC909), and one normal uroepithelial (SV-HUC-1) cell lines. Our results show that cell lines appeared heterogeneous with respect to the expression levels of ProT (Fig. 1f) and HOTAIR (Fig. 1g). Among the bladder cancer cell lines examined, TCCSUP cells relatively expressed the highest levels, whereas J82 and HT1197 cells expressed the lowest levels, of ProT and HOTAIR. Moreover, normal uroepithelial SV-HUC-1 cells expressed relatively low levels of ProT and, in particular, HOTAIR. In line with the data obtained from clinical tumor samples, there was also a positive correlation between the expression levels of ProT and HOTAIR in urothelial cancer cell lines (*r* = 0.7661, *p* = 0.0266) (Fig. 1h). Collectively, these results show that expression of ProT and HOTAIR is upregulated and positively correlated in clinical bladder tumor samples and cancer cell lines.

### Cisplatin treatment increases EGFR and NF-κB activation, as well as ProT and HOTAIR expression in J82 cells, and such effects are abrogated by EGFR inhibitors or ProT knockdown

**Fig. 2:**
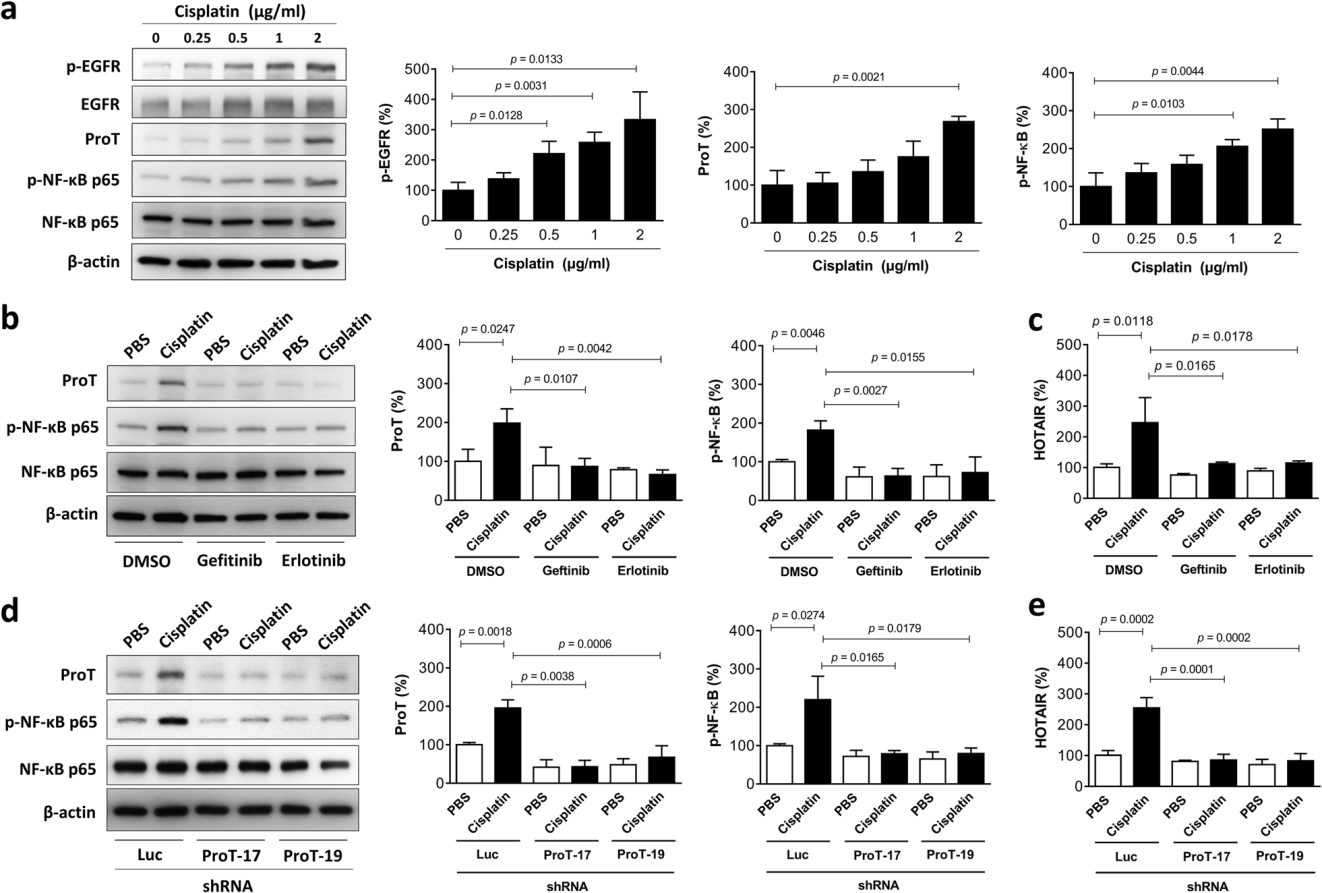
Cisplatin treatment upregulates HOTAIR through the EGFR-ProT axis in bladder cancer cells. **a** Cisplatin treatment activates EGFR and NF-κB and promotes ProT expression. Detection and quantitation of EGFR and its phosphorylated form (p-EGFR), NF-κB p65 and its phosphorylated form (p-NF-κB p65), as well as ProT in J82 cells treated with indicated concentrations of cisplatin (0 to 2 μg/ml) for 48 h by immunoblot analysis. **b, c** Cisplatin treatment activates NF-κB and promotes ProT and HOTAIR expression through the EGFR signaling pathway. J82 cells were treated with cisplatin (2 μg/ml) for 24 h followed by addition of gefitinib (10 μM), erlotinib (1 μM), or the vehicle (DMSO) for an additional 24 h. **d, e** Cisplatin treatment upregulates HOTAIR through ProT. J82 cells that had been transduced with or without lentiviral vectors expressing shRNA specific for human ProT (#17 and #19) or luciferase (Luc) were treated with cisplatin (2 μg/ml) for 48 h. Expression of ProT as well as NF-κB p65 and its phosphorylated form was examined by immunoblot analysis (n = 3) (**b, d**), and HOTAIR was quantified by RT-qPCR (n = 4) (**c, e**). Expression of β-actin served as the loading control for immunoblotting. Representative immunoblots from three independent experiments and quantitative analysis of the indicated proteins are shown (**a, b, d**). Values shown are mean ± SD (n = 4). The ratios of control cells were arbitrarily set to 100

Given that EGFR is not significantly overexpressed in bladder tumors (Fig. 1c) and that cisplatin can induce EGFR activation in various cell types that overexpress the receptor [15], we considered the possibility that cisplatin could induce EGFR activation, leading to upregulation of the proposed ProT-NF-κB-HOTAIR signaling axis and thereby resulting in increases in cachexia-associated pro-inflammatory cytokine production. We treated J82 cells, which endogenously express relatively low levels of ProT and HOTAIR, with cisplatin and detected EGFR activation in terms of phosphorylation. Activation of NF-κB is accompanied by phosphorylation of its subunit p65 (at Ser 536) [50], and the activated NF-κB, in turn, translocates into the nucleus to activate a variety of pro-inflammatory gene expression. Therefore, we examined the expression of ProT and HOTAIR, as well as NF-κB phosphorylation in cisplatin-treated J82 cells. Immunoblot analysis revealed that treatment with cisplatin increased the expression of phospho-EGFR (Tyr1068), ProT, and phospho-NF-κB p65 (Ser536) in a dose-dependent manner (Fig. 2a). To confirm that the activation of EGFR acts upstream of the ProT-NF-κB-HOTAIR signaling axis, the two EGFR kinase inhibitors gefitinib and erlotinib were used to treat J82 cells in the presence or absence of cisplatin. Our results show that treatment with gefitinib or erlotinib reversed cisplatin-induced upregulation of ProT and phospho-NF-κB p65 proteins (Fig. 2b), as well as HOTAIR expression (Fig. 2c). In addition, we used lentivirus-mediated delivery of shRNA specific to ProT (shProT-17 and -19) as well as a control shRNA (shLuc) in J82 cells to further identify ProT as an upstream regulator of the NF-κB-HOTAIR signaling axis. Immunoblot analysis shows that cisplatin-induced NF-κB phosphorylation was abolished in ProT-knockdown J82 cells (Fig. 2d). Notably, cisplatin-induced upregulation of HOTAIR was also abrogated in ProT-knockdown cells (Fig. 2e). Taken together, these results indicate that cisplatin can upregulate the expression of ProT and HOTAIR and that activation of EGFR and NF-κB participates in this pathway in bladder cancer cells. In addition, cisplatin treatment upregulates HOTAIR through the EGFR-ProT axis.

### Overexpression of ProT increases, whereas knockdown of ProT decreases, HOTAIR expression in bladder cancer cells, and HOTAIR upregulation is diminished by treatment with NF-κB inhibitors

**Fig. 3:**
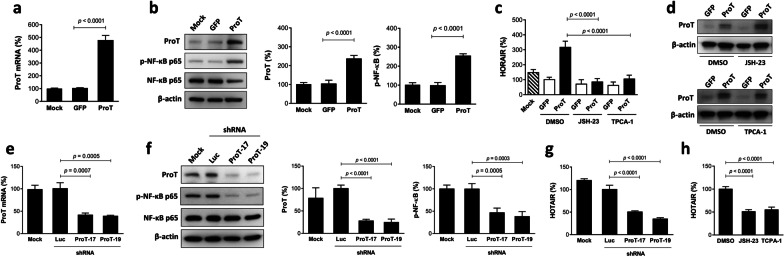
ProT upregulates HOTAIR through NF-κB activation in bladder cancer cells. **a–d** ProT overexpression activates NF-κB and upregulates HOTAIR expression. J82 cells transduced with or without lentiviral vectors expressing ProT (J82/ProT) or vector control GFP (J82/GFP) were examined for ProT transcripts by RT-qPCR (**a**) and levels of ProT as well as NF-κB p65 and its phosphorylated form (p-NF-κB p65) by immunoblotting (**b**). **c, d** J82/ProT and J82/GFP cells were treated with JSH-23 (1 μM), TPCA-1 (1 μM), or the vehicle (DMSO) for 48 h, and levels of HOTAIR and ProT transcripts were assessed by RT-qPCR (**c**) and immunoblotting (**d**), respectively. **e–g** ProT knockdown reduces NF-κB activation and downregulates HOTAIR expression. TCCSUP cells transduced with or without lentiviral vectors expressing shRNA specific for human ProT (#17 and #19) or luciferase (Luc) were examined for ProT (n = 3–4) (**e**) and HOTAIR (**g**) transcripts by RT-qPCR as well as levels of ProT, NF-κB p65, and its phosphorylated form by immunoblotting (**f**). Representative immunoblots from three independent experiments and quantitative analysis of the indicated proteins are shown (**b, f**). **h** TCCSUP cells were treated with JSH-23 (1 μM), TPCA-1 (1 μM), or the vehicle (DMSO) for 48 h, and their HOTAIR levels were assessed by RT-qPCR. Values shown are mean ± SD (n = 3–4). The ratios of control cells were arbitrarily set to 100

Having shown that ProT-knockdown J82 cells did not induce NF-κB phosphorylation (Fig. 2d) and HOTAIR upregulation (Fig. 2e) upon cisplatin treatment, we further overexpressed ProT in J82 cells (J82/ProT) to examine its effects on NF-κB and HOTAIR expression. As expected, J82/ProT cells expressed higher levels of ProT mRNA (Fig. 3a) and protein (Fig. 3b), whereas ProT expression in cells transduced with GFP and parental (mock) cells was at similar levels. In accordance with the result from ProT-knockdown J82 cells (Fig. 2d), overexpression of ProT increased NF-κB phosphorylation (Fig. 3b) and HOTAIR expression (Fig. 3c) in J82 cells. Given that ProT can induce NF-κB activation and that NF-κB can upregulate HOTAIR expression during cisplatin-induced DNA damage [25], we further used NF-κB inhibitors to investigate whether NF-κB signaling was required for the effect of ProT on HOTAIR expression. As shown in Fig. 3c, JSH-23, which inhibits the nuclear translocation of NF-κB p65 and thus abrogates the NF-κB signaling [51] and TPCA-1, an inhibitor of the kinase IKKβ that inhibits activation of the NF-κB signaling pathway [52], could reverse ProT-mediated upregulation of HOTAIR expression in J82 cells. Notably, treatment with these two NF-κB inhibitors did not affect ProT expression, indicating that ProT acts upstream of the NF-κB signaling pathway (Fig. 3d). To further validate that ProT upregulated HOTAIR expression through the NF-κB signaling, we silenced ProT expression in ProT-high-expressing TCCSUP bladder cancer cells via lentivirus-mediated delivery of shRNA specific to ProT, which was confirmed by RT-qPCR (Fig. 3e) and immunoblotting (Fig. 3f). Of note, knockdown of ProT expression decreased NF-κB p65 phosphorylation (Fig. 3f) and HOTAIR expression (Fig. 3g) in TCCSUP cells. Moreover, treatment with NF-κB inhibitors also reduced HOTAIR expression in TCCSUP cells (Fig. 3h), which was similar to the effect of ProT knockdown (Fig. 3g). Collectively, using ProT overexpression and knockdown approaches, we show that inhibition of NF-κB activation abrogates ProT-induced HOTAIR expression, indicating that ProT upregulates HOTAIR expression through activation of the NF-κB signaling pathway. Therefore, on the basis of the data obtained from Figs. 2 and 3, we conclude that cisplatin-induced HOTAIR upregulation is mediated via the EGFR-ProT-NF-κB-HOTAIR signaling axis.

### Overexpression of HOTAIR enhances cell proliferation and pro-inflammatory cytokine expression in J82 cells

**Fig. 4:**
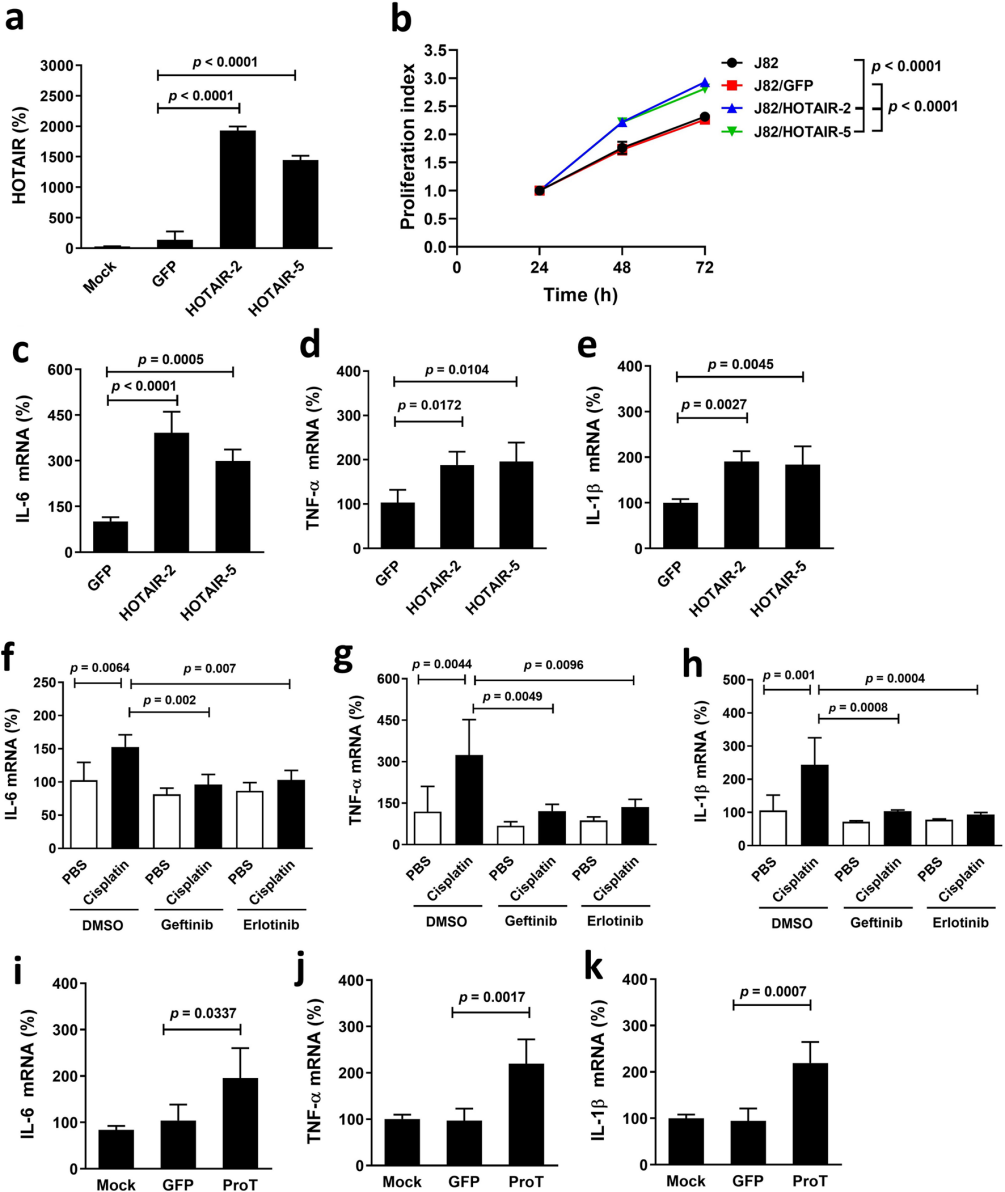
HOTAIR overexpression, cisplatin treatment, and ProT overexpression upregulate pro-inflammatory cytokine expression in human J82 bladder cancer cells. **a–e** J82 cells transduced with or without lentiviral vectors expressing HOTAIR (J82/HOTAIR-2 and -5) or vector control GFP (J82/GFP) were assessed for HOTAIR expression by RT-qPCR (**a**), cell proliferation by the MTS assay (**b**), as well as IL-6 (**c**), TNF-α (**d**), and IL-1β (**e**) transcripts by RT-qPCR. **f–h** J82 cells were treated with cisplatin (2 μg/ml) for 24 h followed by addition of gefitinib (10 μM), erlotinib (1 μM), or the vehicle (DMSO) for an additional 24 h. Expression of IL-6 (**f**), TNF-α (**g**), and IL-1β (**h**) transcripts were assessed by RT-qPCR. **i–k** J82 cells transduced with or without lentiviral vectors expressing ProT (J82/ProT) or vector control GFP (J82/GFP) were quantified for IL-6 (**i**), TNF-α (**j**), and IL-1β (**k**) transcripts by RT-qPCR. Values shown are mean ± SD (n = 4–6). The ratios of control cells were arbitrarily set to 100

As HOTAIR may play roles in inflammation and immune responses, we investigated further the role of HOTAIR in the expression of pro-inflammatory cytokines that contribute to cancer cachexia. We generated HOTAIR-overexpressing J82 (J82/HOTAIR-2 and -5) cells and their vector control (J82/GFP) cells and compared their proliferation rates and cytokine expression levels. Figure 4a confirms that expression levels of HOTAIR in J82/HOTAIR cells were much higher than those of J82/GFP cells. J82/HOTAIR cells significantly proliferated faster than J82/GFP cells or parental cells (Fig. 4b). Moreover, J82/HOTAIR cells significantly expressed higher levels of IL-6 (Fig. 4c), TNF-α (Fig. 4d), and IL-1β (Fig. 4e) than did J82/GFP cells. Given that cisplatin upregulated HOTAIR expression and HOTAIR enhanced pro-inflammatory cytokine expression in J82 cells, we next determined whether treatment with cisplatin upregulated downstream pro-inflammatory cytokines and whether such upregulation could be reversed by EGFR inhibitors. Treatment with gefitinib or erlotinib abolished cisplatin-induced upregulation of IL-6 (Fig. 4f), TNF-α (Fig. 4g), and IL-1β (Fig. 4h) expression in J82 cells. Since ProT acts as an upstream regulator of the NF-κB/HOTAIR axis (Fig. 3), we verified that J82/ProT cells expressed higher levels of IL-6 (Fig. 4i), TNF-α (Fig. 4j), and IL-1β (Fig. 4k) compared to vector control J82/GFP cells. Taken together, these results indicate that forced expression of HOTAIR or ProT in human bladder cancer cells enhances the expression of IL-6, TNF-α, and IL-1β. Additionally, expression of pro-inflammatory cytokines is further elevated in human bladder cancer cells following cisplatin treatment, which can be abrogated by EGFR inhibitors.

### Knockdown of Hotair reduces cell proliferation and pro-inflammatory cytokine expression in MBT-2 cells

**Fig. 5:**
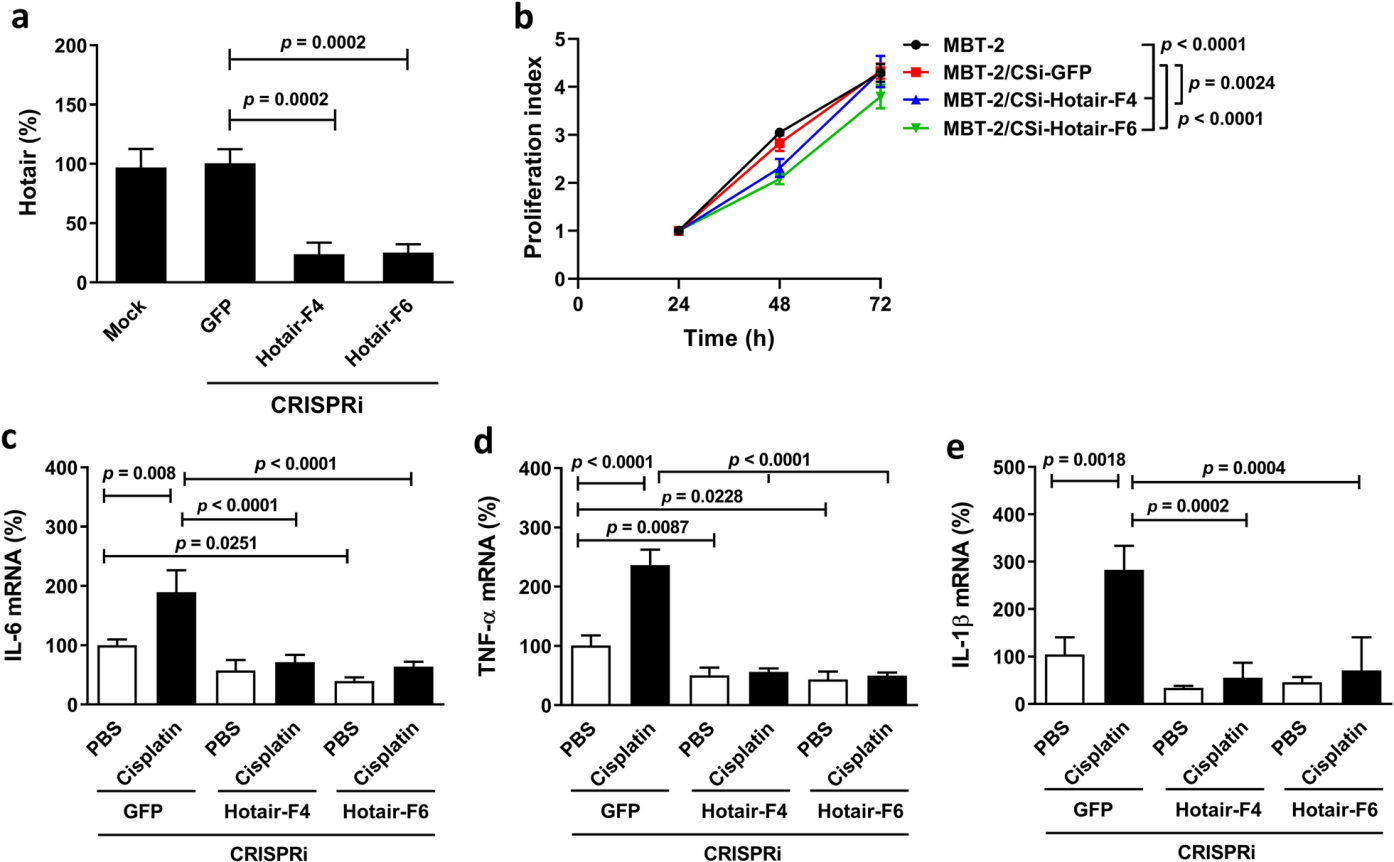
Knockdown of Hotair reduces cell proliferation and downregulates pro-inflammatory cytokine expression in mouse MBT-2 bladder cancer cells. MBT-2 cells transduced with lentiviral vectors containing CRISPRi targeting mouse Hotair (MBT-2/CSi-Hotair-F4 and -F6) or GFP (MBT-2/CSi-GFP) were assessed for Hotair expression by RT-qPCR (**a**), cell proliferation by the MTS assay (**b**), as well as IL-6 (**c**), TNF-α (**d**), and IL-1β (**e**) transcripts by RT-qPCR. Values shown are mean ± SD (n = 3–5). The ratios of control cells were arbitrarily set to 100

Having demonstrated the impact of human HOTAIR on the expression of cachexia-associated pro-inflammatory cytokines in human bladder cancer cells, we sought to confirm whether mouse Hotair had effects similar to human HOTAIR in mouse bladder cancer cells. Since transient transfection of MBT-2 cells with plasmid constructs did not achieve high transfection efficiency, we used lentiviral transduction to mediate the Hotair CRISPRi gene silencing. The transduced cells were selected and expanded under puromycin treatment to obtain MBT-2/CSi-Hotair and vector control MBT-2/CSi-GFP cells. After lentiviral transduction and selection by puromycin, some biological properties between vector control and parental cells may be different. Thus, our knockdown cells were compared with their vector control counterparts rather than parental cells. We tested whether CRISPRi-mediated knockdown of Hotair expression could reduce the expression of downstream pro-inflammatory cytokines in mouse MBT-2 bladder cancer cells that express elevated levels of this lncRNA. Figure 5a shows the confirmation of Hotair knockdown in MBT-2/CSi-Hotair-F4 and -F6 cells compared with MBT-2/CSi-GFP cells. Cell proliferation was slower in HOTAIR-knockdown cells than GFP-knockdown cells or parental cells (Fig. 5b). Moreover, Hotair-knockdown MBT-2 cells expressed lower levels of IL-6 (Fig. 5c), TNF-α (Fig. 5d), and IL-1β (Fig. 5e) compared to GFP-knockdown cells, albeit the difference in IL-1β was not significant (Fig. 5c, d, e). Notably, cisplatin treatment significantly promoted the expression of these cytokines in GFP-knockdown but not Hotair-knockdown cells (Fig. 5c, d, e), indicating that knockdown of Hotair abolished cisplatin-induced upregulation of IL-6, TNF-α, and IL-1β in mouse bladder cancer cells. Collectively, these results demonstrate that knockdown of Hotair decreases cell proliferation and pro-inflammatory cytokine expression and diminishes cisplatin-induced upregulation of pro-inflammatory cytokines in MBT-2 cells. In addition, our results suggest that mouse Hotair has similar biological effects as human HOTAIR on inducing cachexia-associated pro-inflammatory cytokine expression.

### HOTAIR is positively correlated with cisplatin-induced skeletal muscle atrophy in bladder cancer

**Fig. 6:**
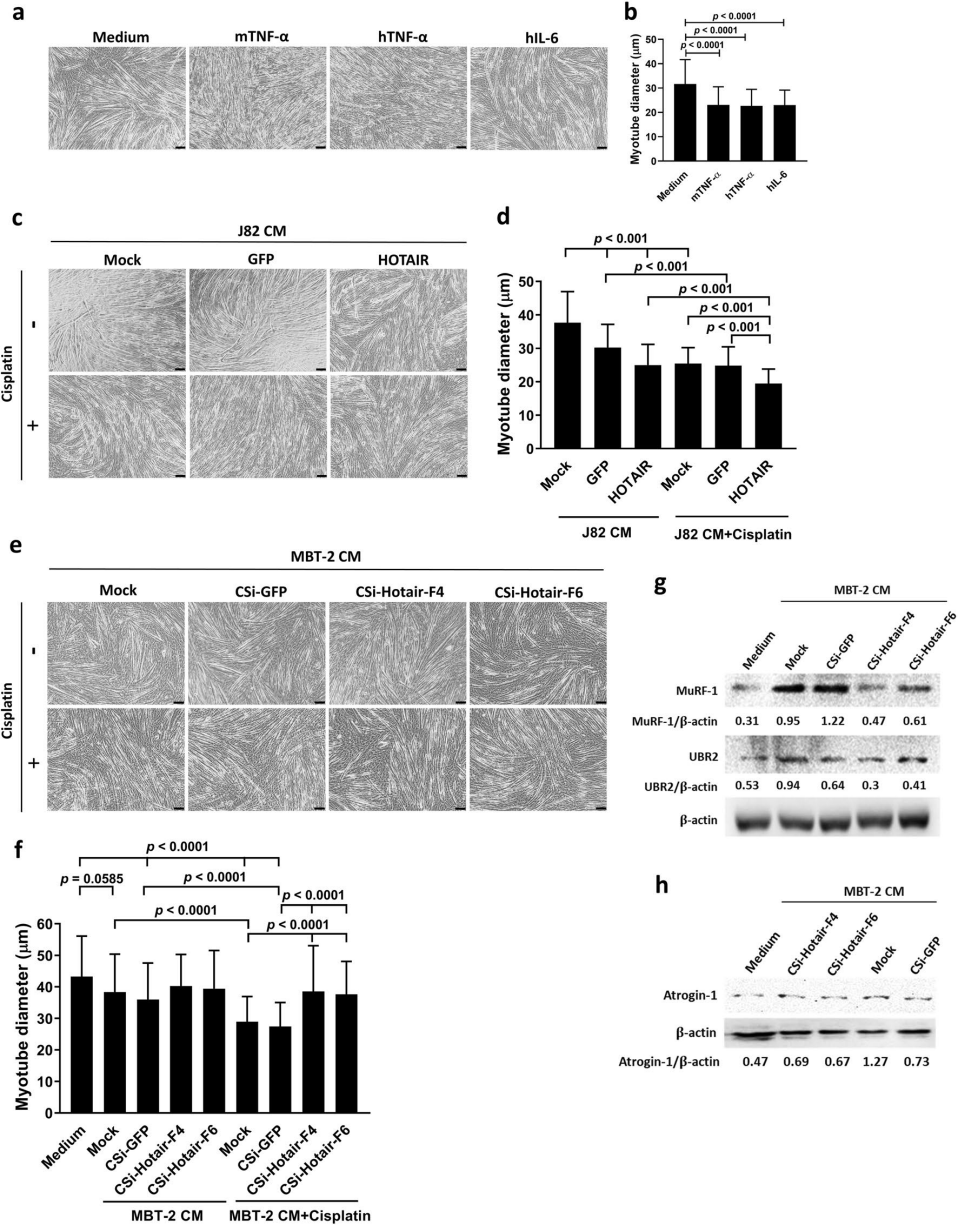
The CM of HOTAIR-overexpressing bladder cancer cells, in particular, in the presence of cisplatin promotes muscle atrophy. Murine C2C12 myoblasts were induced to differentiate into myotubes by culturing the cells in DMEM supplemented with 2% horse serum for 4 days. The CM containing 10% FBS collected from cancer cells was diluted 1:1 with fresh DMEM containing 2% horse serum to treat myotubes for 48 h. **a, b** C2C12 myotubes were treated with 100 ng/ml of mouse TNF-α (mTNF-α), human TNF-α (hTNF-α), or human IL-6 (hIL-6) in DMEM containing 5% FBS and 1% horse serum for 48 h. **c, d** The CM collected from J82/HOTAIR, J82/GFP, or parental (mock) J82 cells in the absence or presence of cisplatin (2 μg/ml) for 48 h was used to treat C2C12 myotubes. **e. f** The CM collected from MBT-2/CSi-Hotair-F4, MBT-2/CSi-Hotair-F6, MBT-CSi-GFP, or parental (mock) MBT-2 cells in the absence or presence of cisplatin (2 μg/ml) for 48 h was used to treat C2C12 myotubes for 48 h. A total of 105 myotubes (15 myotubes/filed) within each section were measured in randomly selected seven different view fields using the ImageJ software to calculate the average diameters. C2C12 representative images (**a, c, e**) and average diameter of myotubes (**b, d, f**). Scale bar = 100 μM. **g, h** Detection of muscle-specific E3 ubiquitin ligases MuRF-1, UBR2, and atrogin-1 in C2C12 myotubes after treatment with the CM of MBT-2 cells or their derivatives by immunoblotting. Expression of β-actin served as the loading control. Values shown below the blots are ratios between the intensity of the bands corresponding to the indicated proteins and those corresponding to β-actin analyzed by densitometry

Given that the most common in vitro muscle model of cancer cachexia is the treatment of murine C2C12 myotubes with the CM collected from cultured cancer cells and that IL-6 and TNF-α are linked to cancer cachexia [5, 53], we next investigated the correlation of HOTAIR expression with skeletal muscle atrophy. C2C12 myotubes were treated with the CM of J82/HOTAIR, J82/GFP, or parental J82 cells in the presence or absence of cisplatin, and muscle atrophy was evaluated by measuring myotube diameters. Recombinant mouse TNF-α was used as a positive control for inducing C2C12 myotube atrophy. Given that pro-inflammatory cytokines contained in the plasma of cachectic patients can induce NF-κB activation in C2C12 cells [3] and human TNF-α and IL-6 are active on both human and murine cells [54, 55], we tested whether the two human cytokines had effects on C2C12 myotubes. Our results confirmed that human TNF-α and IL-6 were capable of inducing mouse myotube atrophy (Fig. 6a, b), indicating that the in vitro murine C2C12 myotube model can be applied for assessing cancer cachexia induced by human cancer cells. The average diameter of C2C12 myotubes exposed to the CM from J82/HOTAIR cells was lower compared with that exposed to the CM from vector control cells (Fig. 6c, d). Moreover, cisplatin treatment further reduced the average diameter of C2C12 myotubes exposed to J82 cell-derived CM (Fig. 6c, d). Apart from examining the CM of human J82 cells after HOTAIR overexpression, we also assessed the impact of murine MBT-2 cell-derived CM after CRISPRi knockdown of Hotair on C2C12 myotube atrophy. In agreement with the data obtained from J82 cells (Fig. 6c, d), treatment with the CM of GFP-knockdown MBT-2 cells significantly decreased the average diameter of C2C12 myotubes compared with the medium control (Fig. 6e, f). Of note, knockdown of Hotair restored the myotube diameter to a similar level of the diameter of control cells exposed to the medium control. In addition, cisplatin treatment resulted in further reduction of the myotube diameter in each treatment. However, knockdown of Hotair in MBT-2 cells alleviated the reduction of myotube diameter induced by the CM of MBT-2 cells exposed to cisplatin. The results from the C2C12 myotube assay suggest that human and mouse bladder cancer cells produce factors to promote skeletal muscle atrophy, in particular, in the presence of cisplatin. Muscle-specific E3 ubiquitin ligases MuRF-1, UBR2 (also known as E3α-II), and atrogin-1 play a key role in mediating the degradation of muscle proteins in cancer cachexia [56, 57]. Therefore, we further examined the expression of these E3 ubiquitin ligases in C2C12 myotubes that had been treated with the CM of MBT-2 cells or their derivatives. Immunoblot analysis shows that the expression of MuRF-1, UBR2, and atrogin-1 was increased in cells treated with the CM of MBT-2 or MBT-2/CSi-GFP cells. Notably, knockdown of Hotair alleviated the upregulation of MuRF-1, UBR2, and atrogin-1 induced by the CM of MBT-2 cells (Fig. 6g, h). Taken together, using C2C12 myotube assay, we demonstrate that HOTAIR plays a pivotal role in cisplatin-induced skeletal muscle atrophy, which may be mediated through upregulation of atrophy-related E3 ubiquitin ligases.

We further investigated whether C2C12 cells also expressed cachexia-associated pro-inflammatory cytokines upon stimulation with the CM of MBT-2 cells in the presence or absence of cisplatin. Additional file 1: Fig. S2 shows that cisplatin treatment enhanced MBT-2 CM-induced expression of IL-6 (Additional file 1: Fig. S2a), TNF-α (Additional file 1: Fig. S2b), and IL-1β (Additional file 1: Fig. S2c) in C2C12 cells. This result combined with results shown above (Figs. 4c-e, 5c-e) suggest that cisplatin promotes bladder cancer-induced skeletal muscle atrophy through upregulation of pro-inflammatory cytokine expression by both tumor cells and skeletal muscle cells.

### Knockdown of Hotair in bladder tumor-bearing mice attenuates cisplatin-induced cancer cachexia

**Fig. 7:**
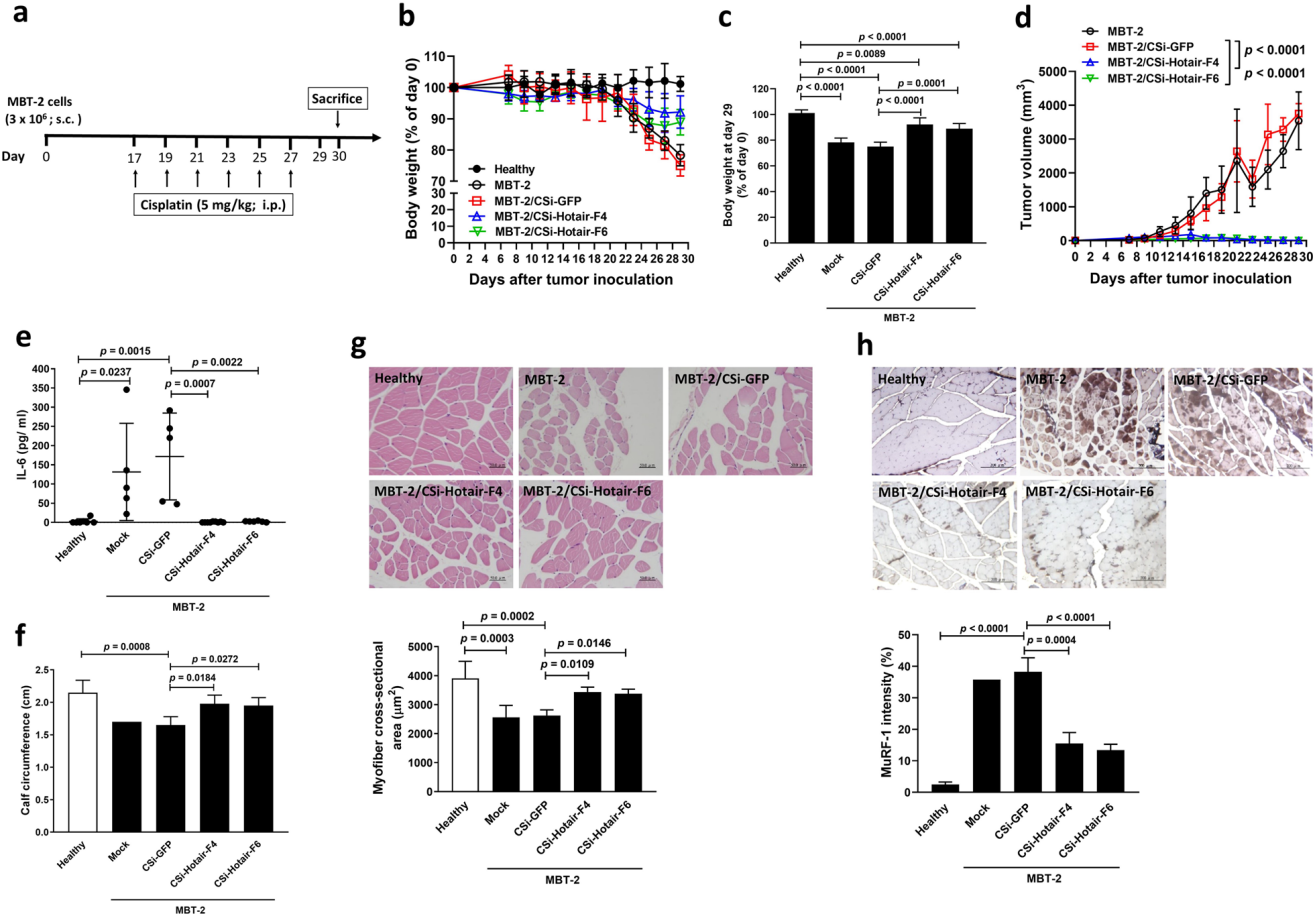
Knockdown of Hotair in MBT-2 bladder tumor-bearing mice attenuates cisplatin-induced cancer cachexia. **a** Treatment schedule. Groups of male C3H/HeN mice were subcutaneously inoculated with 3 × 10^6^ of MBT-2, HOTAIR-knockdown MBT-2 (MBT-2/CSi-Hotair-F4 and -F6), or GFP-knockdown MBT-2 (MBT-2/CSi-GFP) cells at day 0. Another group of mice that received vehicle treatment served as the healthy control. Except for healthy control mice, all mice were treated intraperitoneally with cisplatin (5 mg/kg/day) at days 17, 19, 21, 23, 25 and 27. The mice were sacrificed at day 30. **b** Body weight (after subtraction of the estimated tumor weight, which was estimated as 1 mm^3^ tumor volume equal to 1 mg) was expressed as a percentage of the body weight at day 0. **c** Body weight at day 29 (after subtraction of the absolute tumor weight) was expressed as a percentage of the body weight at day 0. **d** Tumor volumes were measured every two days. Values shown are mean ± SD (n = 4–6) (**b–d**). **e** Serum levels of IL-6 was quantified by ELISA (n = 5–9). **f** Calf circumference was measured at day 30 before sacrifice (n = 2–6). **g** Cross-sectional areas of H&E-stained gastrocnemius muscle tissues (upper) and quantitation using the ImageJ software (lower) (n = 3–6). Scale bar = 50 μm. **h** Immunohistochemistry (upper) and quantitation (lower) of MuRF-1 in muscle tissue sections (n = 2–6). Scale bar = 200 μm

To confirm the relevance of in vitro results, we used the syngeneic MBT-2 bladder tumor model to investigate whether knockdown of Hotair expression in mouse tumors could ameliorate body weight loss and skeletal muscle atrophy in tumor-bearing mice receiving cisplatin chemotherapy. The protocol timeline is shown in Fig. 7a. Body weight loss (after subtraction of the estimated tumor weight) was noted in all groups of mice implanted with MBT-2 cells (Fig. 7b). The relative body weight change (body weight after subtraction of the absolute tumor weight/body weight on day 0) was calculated after sacrifice of the mice at the end of the experiment. While mice bearing MBT-2 or MBT-2/CSi-GFP tumors experienced body weight loss for more than 20%, knockdown of Hotair in MBT-2 tumors significantly improved weight loss in tumor-bearing mice compared to those bearing MBT-2/CSi-GFP tumors at day 29 (Fig. 7c). Notably, much smaller tumor volumes (Fig. 7d), lower serum IL-6 levels (Fig. 7e) comparable to those of healthy mice, and larger calf circumference (Fig. 7f) were detected in MBT-2/CSi-Hotair-bearing mice than in MBT-2/CSi-GFP-bearing mice. With regard to skeletal muscle atrophy, myofiber cross-sectional areas of the gastrocnemius muscle from MBT-2/CSi-Hotair-bearing mice were significantly thicker when compared with those from MBT-2/CSi-GFP cells (Fig. 7g). While the muscle tissues obtained from mice bearing parental MBT-2 or MBT-2/CSi-GFP tumors expressed elevated levels of MuRF-1, its expression was markedly reduced in the muscle tissues from mice bearing MBT-2/CSi-Hotair tumors (Fig. 7h). Altogether, these results demonstrate that cisplatin treatment can induce cachexia in bladder tumor-bearing mice. More importantly, silencing of Hotair expression in bladder tumors can ameliorate cisplatin-induced cachexia in mice. In conclusion, our animal studies provide evidence to show that HOTAIR is involved in cisplatin-induced bladder cancer cachexia.

In addition to MBT-2 tumor implanted in C3H/HeN mice, the MB49 bladder tumor implanted in C57/BL6 mice is widely used as a syngeneic bladder tumor model. Since cachexia found in cancer patients undergoing chemotherapy can be induced by both cancer per se and chemotherapy, we further examined whether cisplatin treatment affected tumor growth and body weight of MB49 bladder tumor-bearing mice (Additional file 1: Fig. S3a). MB49 bladder tumor (MB49/shLuc encoding luciferase shRNA, a vector control shRNA)-bearing C57BL/6 mice treated with cisplatin significantly lost body weight compared with those treated with PBS (Additional file 1: Fig. S3b). Of note, cisplatin treatment significantly reduced tumor volumes of MB49 tumor-bearing mice (Additional file 1: Fig. S3c). Thus, although cisplatin can inhibit tumor growth, it can also induce marked body weight loss, which is one of the common characteristics of cachexia.

### The EGFR inhibitor erlotinib inhibits tumor growth but fails to alleviate cisplatin-induced cancer cachexia in bladder tumor-bearing mice

**Fig. 8:**
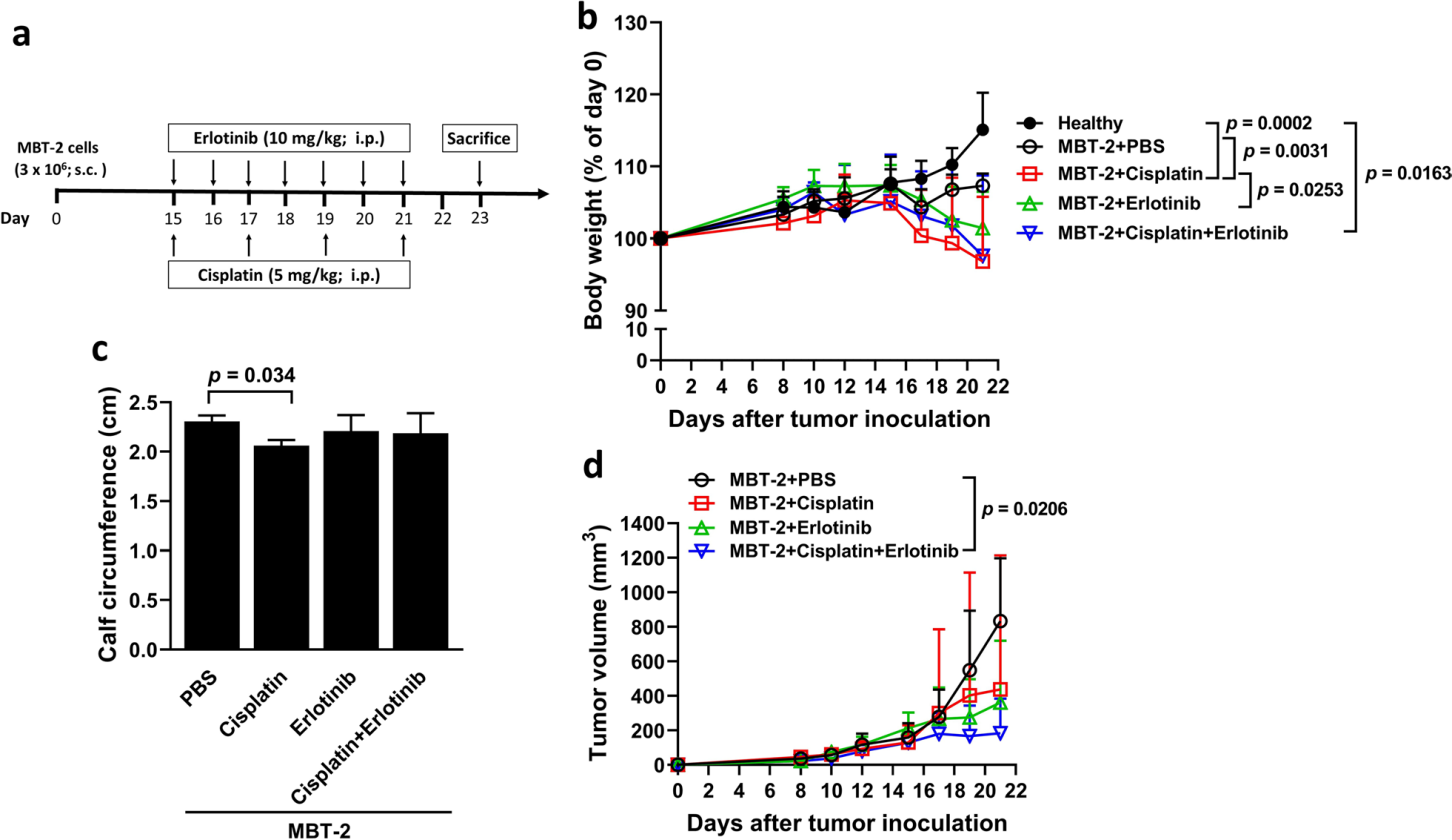
Treatment with erlotinib inhibits tumor growth but fails to alleviate cisplatin-induced cancer cachexia in MBT-2 bladder tumor-bearing mice. **a** Treatment schedule. Groups of male C3H/HeN mice were subcutaneously inoculated with 3 × 10^6^ of MBT-2 cells at day 0. The mice were intraperitoneally treated with erlotinib (10 mg/kg/day) daily from day 15 to day 21 in combination with cisplatin (5 mg/kg/day) at days 15, 17, 19, and 21 and sacrificed at day 23. **b** Body weight (after subtraction of the estimated tumor weight, which was estimated as 1 mm^3^ tumor volume equal to 1 mg) was expressed as a percentage of the body weight at day 0. **c** Calf circumference was measured at day 23 before sacrifice. **d** Tumor volumes were measured every 2 or 3 days. Values shown are mean ± SD (n = 6)

Given the abolishment of cisplatin-induced upregulation of pro-inflammatory cytokines in bladder cancer cells by gefitinib and erlotinib (Fig. 4f, g, h), we next asked whether erlotinib treatment could restore cisplatin-induced cachexia in vivo. The treatment protocol in the MBT-2 tumor model is shown in Fig. 8a. The body weight between healthy mice and MBT-2-bearing mice without receiving either cisplatin or erlotinib did not significantly differ (Fig. 8b). Nevertheless, tumor-bearing mice receiving cisplatin but not erlotinib treatment had lower body weight compared with those receiving the vehicle (PBS). Notably, erlotinib treatment did not improve the body weight loss of tumor-bearing mice treated with cisplatin. Furthermore, there was a reduction in the calf circumference in tumor-bearing mice treated with cisplatin, which could not be restored by erlotinib treatment (Fig. 8c). Regarding tumor size, combination treatment with cisplatin and erlotinib significantly reduced tumor volumes of the mice, whereas single treatment with either cisplatin or erlotinib had no effects on inhibiting tumor growth (Fig. 8d). Taken together, erlotinib treatment in combination with cisplatin in bladder tumor-bearing mice significantly reduces tumor size compared to single treatment with cisplatin or erlotinib. Moreover, our results indicate that cisplatin treatment induces body weight loss and that erlotinib has no effects on alleviating body weight loss and skeletal muscle wasting in bladder tumor-bearing mice.

## Discussion

**Fig. 9:**
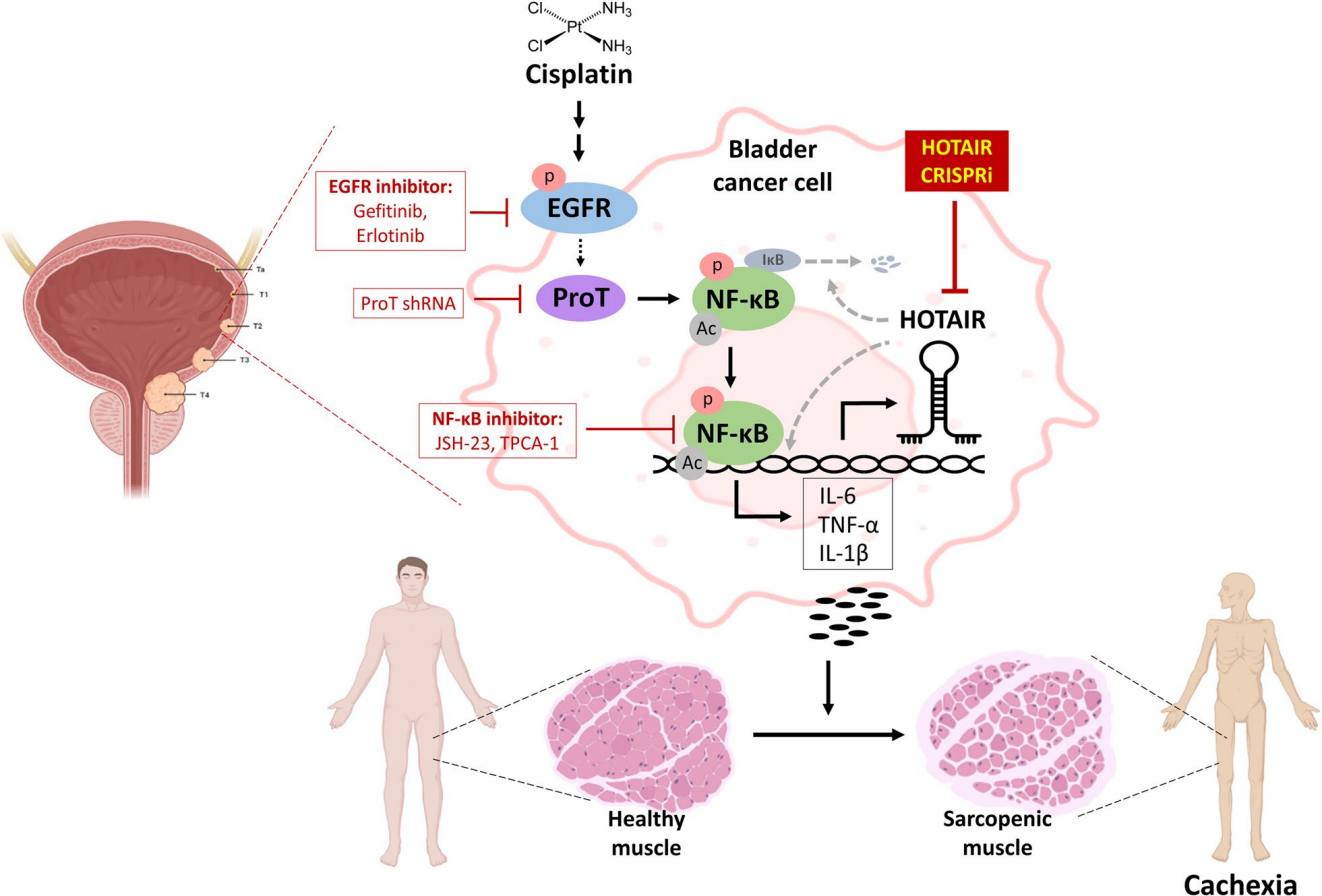
A schematic representation of the EGFR/ProT/NF-κB/HOTAIR axis involved in cisplatin-induced cancer cachexia. In bladder cancer patients receiving cisplatin chemotherapy, cisplatin induces EGFR phosphorylation and activation, leading to upregulation of ProT expression. Excess ProT enhances NF-κB phosphorylation and acetylation [30], thereby increasing its transcriptional activity. Phosphorylated NF-κB translocates to the nucleus, where it binds to the promoter region of HOTAIR and transactivates HOTAIR expression. In addition, HOTAIR facilitates IκBα degradation and thus enhances NF-κB activation, thereby creating a feed-forward regulatory circuit between NF-κB and HOTAIR [25]. Subsequently, excess HOTAIR upregulates expression of the pro-inflammatory cytokines IL-6, TNF-α, and IL-1β, resulting in cancer cachexia. We used EGFR inhibitors (gefitinib and erlotinib), NF-κB inhibitors (JSH-23 and TPCA-1), human ProT overexpression and knockdown, as well as human HOTAIR overexpression and CRISPRi-mediated mouse Hotair knockdown to dissect this signaling axis. The pathways in gray color have been reported previously

In the present study, we demonstrate for the first time that overexpression of HOTAIR contributes to cisplatin-induced bladder cancer cachexia. Using overexpression and knockdown approaches and pharmacological inhibitors, we identify a novel molecular mechanism involving the EGFR-ProT-NF-κB-HOTAIR signaling axis in cisplatin-associated cancer cachexia in bladder cancer cells (Fig. 9). Our results also suggest that the components of this signaling axis may be potential therapeutic targets for bladder cancer patients undergoing cisplatin chemotherapy.

In EGFR signaling pathways, EGF binding to EGFR triggers homodimerization or heterodimerization of this receptor with other ErbB members as well as receptor phosphorylation and activation of downstream effectors, such as RAS-RAF-MEK-ERK-MAPK and PI3K-mTOR, thereby leading to cell proliferation [58]. Because these pathways are interconnected, the enhanced EGFR activity stimulates nearly an entire signaling network and is linked to multiple biological outcomes. This mechanism explains why cisplatin can trigger JNK and p38 activation in other studies [59–61]. As JNK can upregulate ProT in hepatocellular carcinoma cells [62], treatment with a JNK inhibitor decreases the stability of ProT and increases their susceptibility to cisplatin [62]. In the current study, we show that cisplatin induces EGFR activation, thereby resulting in ProT overexpression in bladder cancer cells. However, the mechanistic link between EGFR and ProT is unclear. MicroRNA-1 (miR-1) has been suggested as a tumor suppressor [63]. The expression of miR-1 is downregulated in almost all human tumors examined, including bladder cancer [64]. In addition, miR-1 is a muscle-specific miRNA and plays an essential role in almost every stage of skeletal muscle development [65]. It was shown that nuclear EGFR directly and transcriptionally regulates miR-1 in prostate cancer cells, indicating that miR-1 is one of the targets of EGFR [66]. Moreover, miR-1 was reported to downregulate ProT mRNA expression by directly binding to the 3’-UTR of ProT mRNA in nasopharyngeal carcinoma cells, indicating that ProT is one of miR-1 target genes [67]. Therefore, we proposed that cisplatin-induced EGFR activation can upregulate ProT expression via downregulation of miR-1 in bladder cancer cells.

Our current study from clinical bladder tumor samples indicates significant upregulation of ProT in bladder tumors compared with the corresponding adjacent normal tissues (*p* = 0.013; Fig. 1a) and a positive correlation between the expression levels of ProT and HOTAIR (Fig. 1d). There were trends towards increased expression of HOTAIR (Fig. 1b) and EGFR (Fig. 1c) in tumor tissues compared to adjacent normal tissues. However, due to small sample size (*n* = 6), they did not reach statistical significance. By analyzing TCGA data from 408 bladder cancer patients [48], we found a positive correlation between ProT and HOTAIR (*r* = 0.108, *p* = 0.029) (Fig. 1e) but not between EGFR and HOTAIR (*r* = − 0.039, *p* = 0.434) nor between EGFR and ProT (*r* = − 0.065, *p* = 0.191) (Additional file 1: Fig. S1). In the EGFR signaling pathway, interaction of EGFR extracellular domain with its ligands, such as EGF or other HER family members, induces homodimerization or heterodimerization that causes activation of the tyrosine kinase domain resulting in tyrosine autophosphorylation to recruit adaptor proteins, leading to activating multiple signaling pathways and subsequently regulating several intracellular processes, such as proliferation, cellular repair, protection from injury, and anti-apoptosis [68]. Thus, the phospho-EGFR rather than total EGFR is more representative of activated EGFR. Herein, we show that treatment with cisplatin dose-dependently increased the expression of phospho-EGFR but not EGFR, with concomitant upregulation of ProT and phospho-NF-κB in J82 cells (Fig. 2a). Furthermore, the EGFR inhibitors gefitinib and erlotinib could abrogate cisplatin-induced upregulation of ProT, phospho-NF-κB, and HOTAIR (Fig. 2b). These results highlight the importance of EGFR activation in cisplatin-induced HOTAIR upregulation. Gefitinib, erlotinib, and afatinib have been approved for lung cancer treatment as a first-line therapy in those cases with EGFR mutations [69]. Regarding bladder cancer, a phase II trial has shown that erlotinib used as a neoadjuvant, when administered before radical cystectomy, had beneficial effects in terms of surgical pathology and short-term clinical outcomes in patients with invasive bladder cancer [70]. EGFR family inhibitors have been suggested to be useful in muscle invasive bladder cancer patients with no prior chemotherapy in whom EGFR or ErbB2 is overexpressed [71].

Therapies targeting the EGFR signaling, such as small-molecule tyrosine kinase inhibitors (TKIs), have been used to treat a variety of cancers. In the current study, we demonstrate that cisplatin treatment induces EGFR activation in bladder cancer cells, resulting in the upregulation of the ProT-NF-κB-HOTAIR axis and leading to promoting cachexia-associated pro-inflammatory cytokine expression. One would expect that treatment with erlotinib or gefitinib would alleviate cachexia symptoms associated with cisplatin chemotherapy. In the present study, we treated MBT-2 tumor-bearing mice with cisplatin and/or erlotinib and evaluated their anti-tumor and anti-cachexic effects (Fig. 8). Despite its antitumor effect when combined with cisplatin, consecutive intraperitoneal treatment with erlotinib (10 mg/kg/day) for 7 days to tumor-bearing mice (mean tumor volume of 157 mm^3^) failed to alleviate cisplatin-induced cachexia (Fig. 8b, c). Notably, cachexia was caused by cisplatin but not MBT-2 tumor per se, which was also confirmed in the MB49 bladder tumor model (Additional file 1: Fig. S3). In the Lewis lung carcinoma (LLC)-induced cachexia model in C57BL/6 mice with a similar treatment regimen of erlotinib as ours, erlotinib treatment ameliorated cancer cachexia in tumor-bearing mice [72]. Notably, the LLC-bearing mice did not receive any chemotherapy. In a retrospective study on non-small-cell lung cancer (NSCLC) patients undergoing either cytotoxic chemotherapy with platinum-based drugs or molecular targeted therapy with TKIs of EGFR or anaplastic lymphoma kinase, skeletal muscle loss was lower in patients receiving TKIs than in those receiving cytotoxic chemotherapy, suggesting that TKIs may be less prone to induce cancer cachexia than cytotoxic chemotherapy [73]. However, given that TKIs target enzymes that participate in the activation of intracellular signaling pathways, such as the PI3K-AKT-mTOR pathway, which also regulates muscle protein synthesis [74], muscle wasting could be exacerbated in cancer patients treated with TKIs [75]. Sarcopenia was highly prevalent in NSCLC patients harboring EGFR mutations and treated with gefitinib [76]. Moreover, 42% of NSCLC patients had skeletal muscle loss during gefitinib or afatinib treatment, which was correlated with poor prognosis [77]. In patients with metastatic colorectal cancer harboring wild-type RAS, skeletal muscle loss during anti-EGFR therapy combined with chemotherapy predicted poor prognosis [78]. Therefore, blockade of EGFR signaling by either small-molecule inhibitors or anti-EGFR monoclonal antibodies may not only inhibit tumor growth but also exacerbate cachexia symptoms in clinical settings. Along this line of speculation, it would be difficult to use erlotinib for validating the involvement of the EGFR-ProT-NF-κB-HOTAIR signaling axis in cisplatin-induced cancer cachexia in mice. Furthermore, since our treatment regimens were not optimized, we could not exclude the possible efficacy of erlotinib on improving cachexia symptoms. Emerging lines of evidence indicates that drug resistance to EGFR inhibitors frequently occurs through multiple, interacting pathways [79]. It was suggested that successful cachexia treatment may require combination of drugs with different mechanisms of action [80].

HOTAIR is a well-studied oncogenic lncRNA in various cancers. Forced overexpression of HOTAIR promotes, whereas constitutive knockdown of HOTAIR reduces, cell proliferation in cancer cells [20, 81]. Herein, our in vitro data demonstrate that HOTAIR positively regulates proliferation of bladder cancer cells (Figs. 4b, 5b). MBT-2/CSi-Hotair cells proliferated slower than MBT-2/CSi-GFP cells in vitro (Fig. 5b). Most notably, in vivo growth of MBT-2/CSi-Hotair tumors in syngeneic mice was almost completely suppressed, in particular after cisplatin treatment starting on day 17, whereas MBT-2/CSi-GFP and parental MBT-2 cells continued to grow despite treatment with cisplatin (Fig. 7d). Regarding cachexia, knockdown of Hotair in MBT-2 tumors ameliorated cisplatin-induced cachexia in terms of changes in body weight (Fig. 7c), serum IL-6 levels (Fig. 7e), muscle mass (Fig. 7f, g), and MuRF-1 expression (Fig. 6h). It was shown that neutralization of human IL-6 by monoclonal antibodies reduced tumor-induced cachexia independent of an effect on tumor growth in human tumor xenograft models in nude mice, supporting a role for tumor-derived IL-6 in cancer cachexia [82]. In patients with resectable pancreatic cancer, tumor-associated cachexia was not necessarily dependent on tumor size or load [83]. In the current study, the improvement of cisplatin-induced cachexia by Hotair knockdown in murine bladder tumors can be attributed to direct effect of Hotair downregulation on reducing pro-inflammatory cytokines associated with cachexia. However, we cannot rule out the possibility that reduced cachexia by Hotair knockdown may, in part, due to decreased tumor size, leading to the reduction of tumor-derived pro-inflammatory cytokine production.

Accumulating evidence has revealed that HOTAIR, which is upregulated in a variety of cancers, contributes to chemoresistance in cancer cells by various mechanisms, such as inhibition of cell apoptosis, dysregulation of cell cycle, enhancements of EMT, autophagy, and self-renewal of cancer stem cells, interference with DNA repair, and alteration of drug efflux pump [84]. Given the role of HOTAIR in chemotherapy resistance, we compared the IC_50_ values of cisplatin in Hotair-knockdown and parental MBT-2 cells. Knockdown of Hotair resulted in an approximately two-fold decrease in the IC_50_ value of cisplatin in MBT-2 cells (Additional file 1: Fig. S4), suggesting that HOTAIR may mediate cisplatin resistance in bladder cancer. It is reasonable to assume that inhibition of MBT-2 tumor growth by Hotair knockdown in mice may be, in part, attributed to increased cisplatin sensitivity. Thus, the EGFR-ProT-NF-κB-HOTAIR may also play a significant role in cisplatin-induced anticancer resistance. However, the mechanism underlying the role of HOTAIR in the resistance to cisplatin in bladder cancer is unclear. Our data obtained from bladder cancer are consistent with previous reports in other cancers showing the involvement of HOTAIR in cisplatin resistance. In small-cell lung cancer, HOTAIR could induce HOXA1 methylation and lead to multidrug resistance through the NF-κB pathway [85, 86]. Moreover, knockdown of HOTAIR inhibited expression of the multidrug-resistance genes ABCB1, ABCC1, and ABCG2 in cisplatin-resistant gastric cancer cells and reduced xenograft tumor growth in nude mice [87]. HOTAIR also participated in the resistance to doxorubicin in bladder cancer cells [88]. In our animal studies, in addition to attenuation of cancer cachexia, knockdown of Hotair dramatically suppressed in vivo growth of MBT-2 tumors (Fig. 7). Moreover, erlotinib inhibited tumor growth but failed to alleviate cancer cachexia in MBT-2 tumor-bearing mice. Since cancer progression and cancer cachexia share overlapping interconnected signaling pathways, the pathophysiological basis of cachexia in cancer is difficult to unravel [89]. Hence, we speculate that the EGFR-ProT-NF-κB-HOTAIR signaling axis contributes to both cisplatin-induced cachexia and tumor growth caused by cisplatin resistance. Nevertheless, the detailed mechanism still awaits further investigation. Previous studies have shown that targeted blockade of cachexia signaling with a novel HDAC inhibitor or a decoy receptor of ActRIIB prolonged survival while tumors continued to grow in animal models of cancer-induced cachexia [90, 91]. Thus, novel approaches or therapeutic targets are warranted for treating cancer cachexia.

In the present study, we used mouse C2C12 myoblasts as an in vitro muscle atrophy model to examine the impact of HOTAIR on myotube atrophy. Human J82 tumor-derived CM reduced C2C12 myotube diameter, and cisplatin treatment led to further reduction (Fig. 6c, d). Notably, HOTAIR-overexpressing J82 cells treated with cisplatin resulted in even further reduction in the myotube diameter. By contrast, knockdown of mouse Hotair had opposite effects (Fig. 6e, f). Thus, we demonstrate that HOTAIR plays a critical role in skeletal muscle atrophy in cisplatin-induced cancer cachexia. We also show that MBT-2 tumor-derived CM upregulated the expression of atrophy-related E3 ubiquitin ligases MuRF-1, UBR2, and atrogin-1 in C2C12 cells, which could be downregulated by Hotair knockdown (Fig. 6g, h). Our results suggest that HOTAIR promotes cachexia-associated pro-inflammatory cytokine expression and enhances the expression of atrophy-related E3 ubiquitin ligases, leading to skeletal muscle atrophy and finally muscle wasting and cachexia.

In our syngeneic MBT-2 bladder tumor model, except for the healthy control group, all the mice inoculated with parental MBT-2 cells or their derivatives were treated with cisplatin (Fig. 7a). We did not include the groups of tumor-bearing mice without cisplatin treatment due to insufficient animal supply. Nevertheless, in another set of the animal experiment using the MB49 bladder tumors, we show that cisplatin treatment induced marked body weight loss in spite of the fact that it also inhibited tumor growth (Additional file 1: Fig. S3). Since we did not have the mouse Hotair expression vector, and MBT-2 cells express elevated levels of Hotair and therefore are not suitable for overexpression experiments, we were unable to manipulate Hotair overexpression in mouse bladder cancer cells for demonstrating the reversibility of animal results using Hotair-knockdown MBT-2 cells (Fig. 7). Nevertheless, using the in vitro C2C12 myotube model, we show that the CM collected from HOTAIR-overexpressing J82 cells promoted, whereas the CM from Hotair-knockdown MBT-2 cells decreased, cisplatin-induced skeletal muscle atrophy (Fig. 6).

It has been documented that lncRNAs show greater conservation of genomic synteny than sequence identity [92]. The mouse Hotair has only 58% sequence identity to human HOTAIR. While human HOTAIR is comprised of 6 exons (1–6 exons), mouse Hotair has 5 exons where exon 2 (human analog) is absent [93]. HOTAIR consists of two regions including rich conserved (i.e. exons 1, 3–5, and domain B of exon 6) and poorly conserved genomic area (exon 2 and domain A of exon 6). Studies on mouse Hotair using genetic knockout mice have yielded contradicting results for the effects and actions of mouse Hotair compared to human HOTAIR. The discrepancy may be attributed to different approaches for target disruption of the Hotair gene and different methods for assays [94, 95]. Since regions important for HOTAIR functions are highly conserved, HOTAIR might have similar functions between mouse and human species. Similar roles for HOTAIR in the regulation of human and mouse *HOXD* genes have been reported [95]. Herein, we overexpressed human HOTAIR in human J82 bladder cancer cells that express low levels of HOTAIR as well as silenced mouse Hotair expression in murine MBT-2 bladder cancer cells that express elevated levels of Hotair. Using the in vitro C2C12 myotube assay, we show that both human HOTAIR and mouse Hotair promote pro-inflammatory cytokine expression, thereby contributing to cisplatin-induced muscle atrophy in bladder cancer cells probably through enhancing the expression of muscle-specific E3 ubiquitin ligases, including MuRF-1, UBR2, and atrogin-1 (Fig. 6). Our results demonstrate that HOTAIR from both human and mouse species plays a pivotal role in cancer cachexia associated with cisplatin chemotherapy, suggesting that they share similar biological functions. Therefore, our results are in accordance with previous studies showing the conservation of HOTAIR in gene synteny and function rather than primary sequence [92].

Cancer cachexia is characterized by progressive skeletal muscle loss and accompanied by increased releases of pro-inflammatory cytokines, which are mainly produced by immune cells in response to cancer. Since IL-6, TNF-α, and IL-1β, and IL-6 receptors are expressed in skeletal muscle [96], skeletal muscle may be regarded as a component of the immune system. In the current study, bladder cancer cells treated with cisplatin expressed higher levels of pro-inflammatory cytokine transcripts compared to untreated cells. However, no detectable levels of IL-6, TNF-α, and IL-1β were found in the CM of J82 cells by ELISA with the detection limits of 9, 15, and 4 pg/ml, respectively. We were also unable to detect these cytokines in the CM of MBT-2 cells. Therefore, we used RT-qPCR to determine relative levels of intracellular cytokine transcripts after cells were treated with cisplatin. Based on the raw data of RT-qPCR, IL-1β and IL-6 transcripts in J82 and MBT-2 cells were the highest, respectively, followed by TNF-α in both cells (data not shown). The microenvironments of cancer cells cultured in vitro and grown in vivo are quite different, which may affect their properties, especially upon various stimuli, such as inflammatory stimuli. As J82 and MBT-2 cells were cultured in DMEM with or without cisplatin, which may be regarded as a relative clean environment, it is unsurprising that they did not secrete detectable cytokines. Some cancer cell lines may naturally secrete pro-inflammatory cytokines, whereas some can be induced to secrete these cytokines. The tumor- and host-produced cytokines, such as IL-6 and TNF-α, are believed to play a significant role in the catabolism and weight loss associated with some malignant and nonmalignant conditions [97, 98].

In the present study, our results highlight for the first time a critical role for HOTAIR in cisplatin-induced bladder cancer cachexia. Our findings also provide insights into the molecular mechanisms underlying cisplatin-induced cachexia in patients with bladder cancer and likely other cancers. The EGFR-ProT-NF-κB-HOTAIR signaling axis is involved in this signaling cascade. Our findings may also provide a mechanistic explanation for why cisplatin may reduce tumor burden but still induce muscle wasting and cachexia in cancer patients undergoing cisplatin chemotherapy. Pharmacological inhibitors of EGFR and NF-κB signaling pathways as well as modulation of ProT and HOTAIR expression may be further explored for ameliorating cachexia in cancer patients undergoing chemotherapy. However, the limitation of our study is the lack of sufficient clinical evidence to validate the positive correlation of ProT and HOTAIR expression with cachexia in cancer patients receiving cisplatin chemotherapy. In addition, the cellular behavior in vitro sometimes is greatly different from their responses in vivo. Although it has proven difficult to accomplish in vivo, the role of the EGFR-ProT-NF-κB-HOTAIR signaling axis in cisplatin-induced cancer cachexia requires validation in animal models. Therefore, these issues warrant further investigations.

## Conclusions

To the best of our knowledge, this is the first report to demonstrate a critical role for HOTAIR in cisplatin-induced cachexia in bladder cancer and to identify HOTAIR as a new therapeutic target for cachexia in bladder cancer and likely other cancers. Although the involvement of other genes or signaling pathways in cisplatin-induced cachexia in bladder cancer cannot be overlooked, the components of the EGFR-ProT-NF-κB-HOTAIR signaling axis represent targets for novel therapeutic strategies to prevent or ameliorate cisplatin-induced cachexia in bladder cancer and other cancers.

## Supplementary Information

**Additional file 1: Figure S1. a, b** There are no correlations between the expression of EGFR and HOTAIR (**a**) and EGFR and ProT (PTMA) (**b)** analyzed from the TCGA bladder cancer cohort (n = 408). **Figure S2.** C2C12 myotubes treated with the conditioned medium (CM) of MBT-2 cells in the presence of cisplatin increase pro-inflammatory cytokine expression. The CM collected from MBT-2 cells that had been treated with cisplatin (2 μg/ml) for 48 h and replenished with fresh medium for an additional 24 h was used to treat C2C12 myotubes for 48 h. Expression of IL-6 (**a**), TNF-α (**b**), and IL-1β (**c**) transcripts were assessed by RT-qPCR. Values shown are mean ± SD (n = 5, 4, and 2 for **a**, **b**, and **c,** respectively; Student’s *t*-test). **Figure S3.** Cisplatin inhibits tumor growth and reduces body weight in MB49 bladder tumor-bearing mice. **a** Treatment schedule. C57BL/6 mice were subcutaneously inoculated with 3 × 10^6^ of MB49/shLuc cells that express shLuc (control shRNA) at day 0 with or without cisplatin treatment (5 mg/kg/day) at days 15, 17, 19, 21, and 23. The observation period ended at day 25. **b** Body weight (after subtraction of the estimated tumor weight, which was estimated as 1 mm^3^ tumor volume equal to 1 mg) was measured every two or three days and expressed as a percentage of the body weight at day 0. **c** Tumor volumes were measured every two or three days. Values shown are mean ± SD (n = 4; two-way ANOVA with repeated measures). **Figure S4.** Knockdown of Hotair enhances sensitivity to cisplatin in MBT-2 cells. Hotair-knockdown MBT-2 cells (MBT-2/CSi-Hotair-F4 and -F6) and parental cells (5 × 10^3^) that had been cultured in 96-well plates overnight were refed with the fresh medium containing various concentrations of cisplatin. After 24 h, cell viability was assessed with the colorimetric WST-8 assay (**a**), and IC_50_ values of cisplatin in different cells are determined (**b**). Values represent the relative cell survival, with the viability in the parental MBT-2 cells without cisplatin treatment arbitrarily set to 100. Values shown are mean ± SD (n = 3).

## Data Availability

All data generated in the present study may be requested from the corresponding authors.

## Abbreviations

CRISPRi (CSi): CRISPR interference
EMT: Epithelial-mesenchymal transition
EGFR: Epidermal growth factor receptor
GPCRs: G-protein-coupled receptors
HDAC: Histone deacetylases
HOTAIR: HOX Transcript Antisense RNA
IC_50_: 50% Inhibitory concentration
IL: Interleukin
LLC: Lewis lung carcinoma
LncRNA: Long non-coding RNA
MiR-1: MicroRNA-1
MuRF1: Muscle RING finger-1
PAR-2: Protease-activated receptor 2
ProT: Prothymosin α
STAT3: Signal transducer and activator of transcription 3
TCGA: The Cancer Genome Atlas
TKIs: Tyrosine kinase inhibitors
TLR4: Toll-like receptor 4
TNF-α: Tumor-necrosis factor α
