# Supplementary material for: Interruption of the long non-coding RNA HOTAIR signaling axis ameliorates chemotherapy-induced cachexia in bladder cancer
Source: J Biomed Sci. 2022 Dec 6;29:104. doi: 10.1186/s12929-022-00887-y (PMC9724340; doi:10.1186/s12929-022-00887-y)
Supplement: Supplementary file 1 — Additional file 1: Figure S1. a, b There are no correlations between the expression of EGFR and HOTAIR (a) and EGFR and ProT (PTMA) (b) analyzed from the TCGA bladder cancer cohort (n = 408). Figure S2. C2C12 myotubes treated with the conditioned medium (CM) of MBT-2 cells in the presence of cisplatin increase pro-inflammatory cytokine expression. The CM collected from MBT-2 cells that had been treated with cisplatin (2 μg/ml) for 48 h and replenished with fresh medium for an additional 24 h was used to treat C2C12 myotubes for 48 h. Expression of IL-6 (a), TNF-α (b), and IL-1β (c) transcripts were assessed by RT-qPCR. Values shown are mean ± SD (n = 5, 4, and 2 for a, b, and c, respectively; Student’s t-test). Figure S3. Cisplatin inhibits tumor growth and reduces body weight in MB49 bladder tumor-bearing mice. a Treatment schedule. C57BL/6 mice were subcutaneously inoculated with 3 × 10^6^ of MB49/shLuc cells that express shLuc (control shRNA) at day 0 with or without cisplatin treatment (5 mg/kg/day) at days 15, 17, 19, 21, and 23. The observation period ended at day 25. b Body weight (after subtraction of the estimated tumor weight, which was estimated as 1 mm3 tumor volume equal to 1 mg) was measured every two or three days and expressed as a percentage of the body weight at day 0. c Tumor volumes were measured every two or three days. Values shown are mean ± SD (n = 4; two-way ANOVA with repeated measures). Figure S4. Knockdown of Hotair enhances sensitivity to cisplatin in MBT-2 cells. Hotair-knockdown MBT-2 cells (MBT-2/CSi-Hotair-F4 and -F6) and parental cells (5 × 10^3^) that had been cultured in 96-well plates overnight were refed with the fresh medium containing various concentrations of cisplatin. After 24 h, cell viability was assessed with the colorimetric WST-8 assay (a), and IC50 values of cisplatin in different cells are determined (b). Values represent the relative cell survival, with the viability in the parental MBT-2 cells without cisplatin treatme [file 12929_2022_887_MOESM1_ESM.docx]

**Supporting information for**

**Interruption of the long non-coding RNA HOTAIR signaling axis ameliorates chemotherapy-induced cachexia in bladder cancer**

Che-Yuan Hu, Bing-Hua Su, Ya-Che Lee, Chung-Teng Wang, Mei-Lin Yang, Wan-Ting Shen, Jing-Ting Fu, Shih-Yao Chen, Wei-Yun Huang, Chien-Hui Ou, Yuh-Shyan Tsai, Feng-Chih Kuo, Ai-Li Shiau, Gia-Shing Shieh, Chao-Liang Wu

**
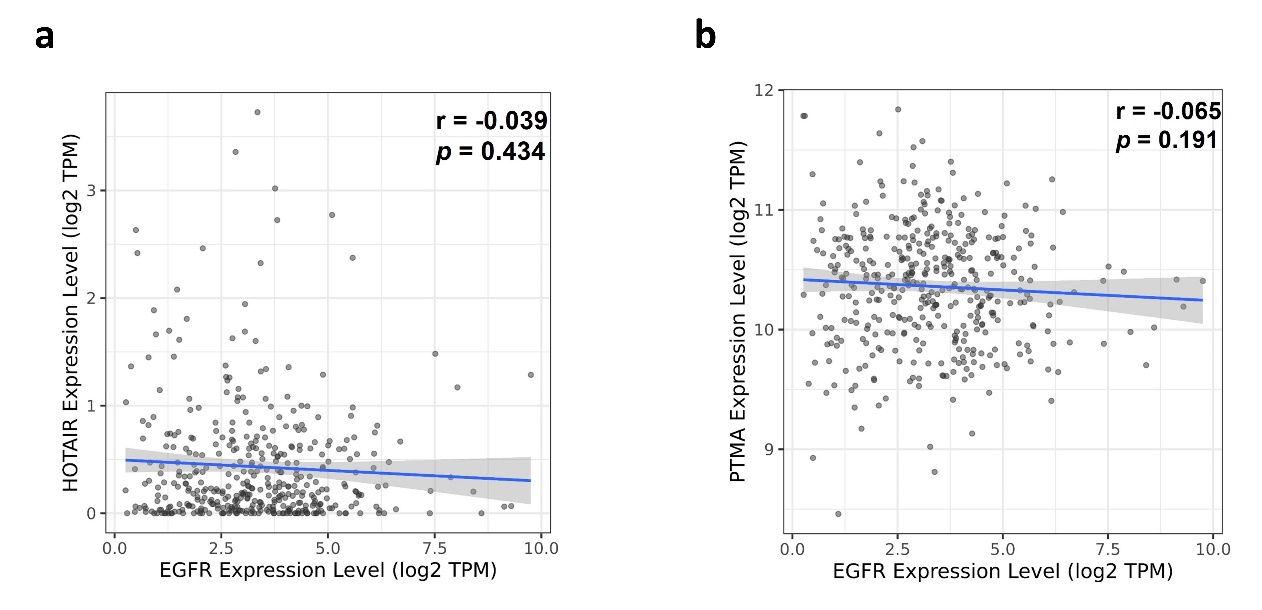
**

**Fig. S1 a, b** There are no correlations between the expression of EGFR and HOTAIR (**a**) and EGFR and ProT (PTMA) (**b)** analyzed from the TCGA bladder cancer cohort (n = 408).


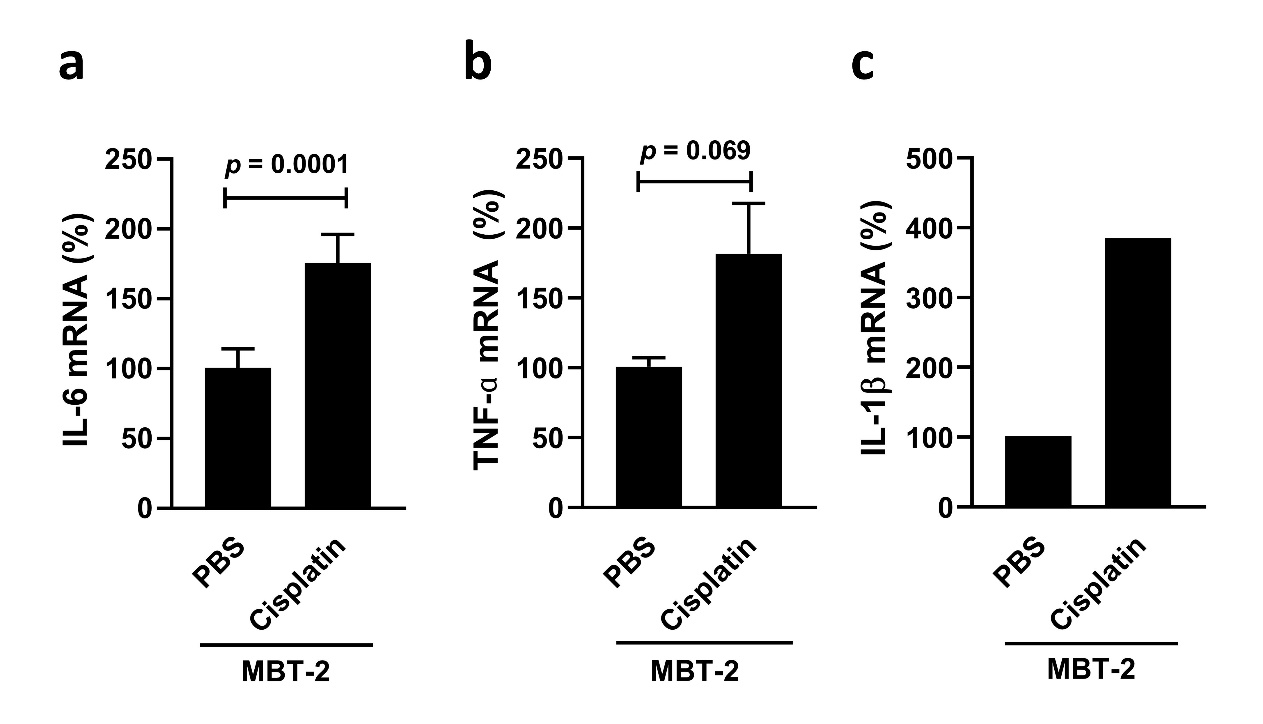


**Fig. S2** C2C12 myotubes treated with the conditioned medium (CM) of MBT-2 cells in the presence of cisplatin increase pro-inflammatory cytokine expression. The CM collected from MBT-2 cells that had been treated with cisplatin (2 μg/ml) for 48 h and replenished with fresh medium for an additional 24 h was used to treat C2C12 myotubes for 48 h. Expression of IL-6 (**a**), TNF-α (**b**), and IL-1β (**c**) transcripts were assessed by RT-qPCR. Values shown are mean ± SD (n = 5, 4, and 2 for **a**, **b**, and **c,** respectively; Student’s *t*-test).

**
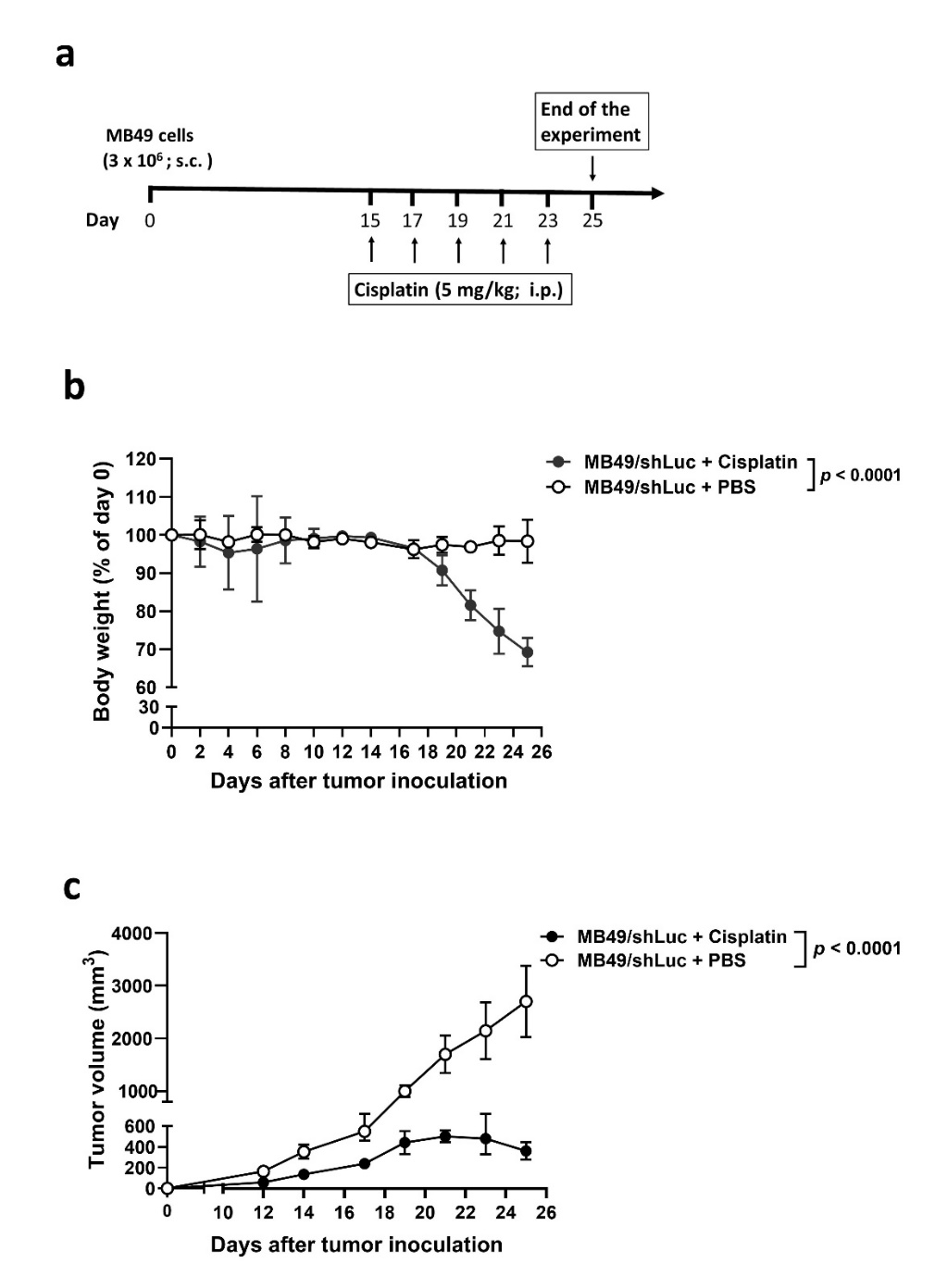
Fig. S3** Cisplatin inhibits tumor growth and reduces body weight in MB49 bladder tumor-bearing mice. **a** Treatment schedule. C57BL/6 mice were subcutaneously inoculated with 3 × 10^6^ of MB49/shLuc cells that express shLuc (control shRNA) at day 0 with or without cisplatin treatment (5 mg/kg/day) at days 15, 17, 19, 21, and 23. The observation period ended at day 25. **b** Body weight (after subtraction of the estimated tumor weight, which was estimated as 1 mm^3^ tumor volume equal to 1 mg) was measured every two or three days and expressed as a percentage of the body weight at day 0. **c** Tumor volumes were measured every two or three days. Values shown are mean ± SD (n = 4; two-way ANOVA with repeated measures).

**
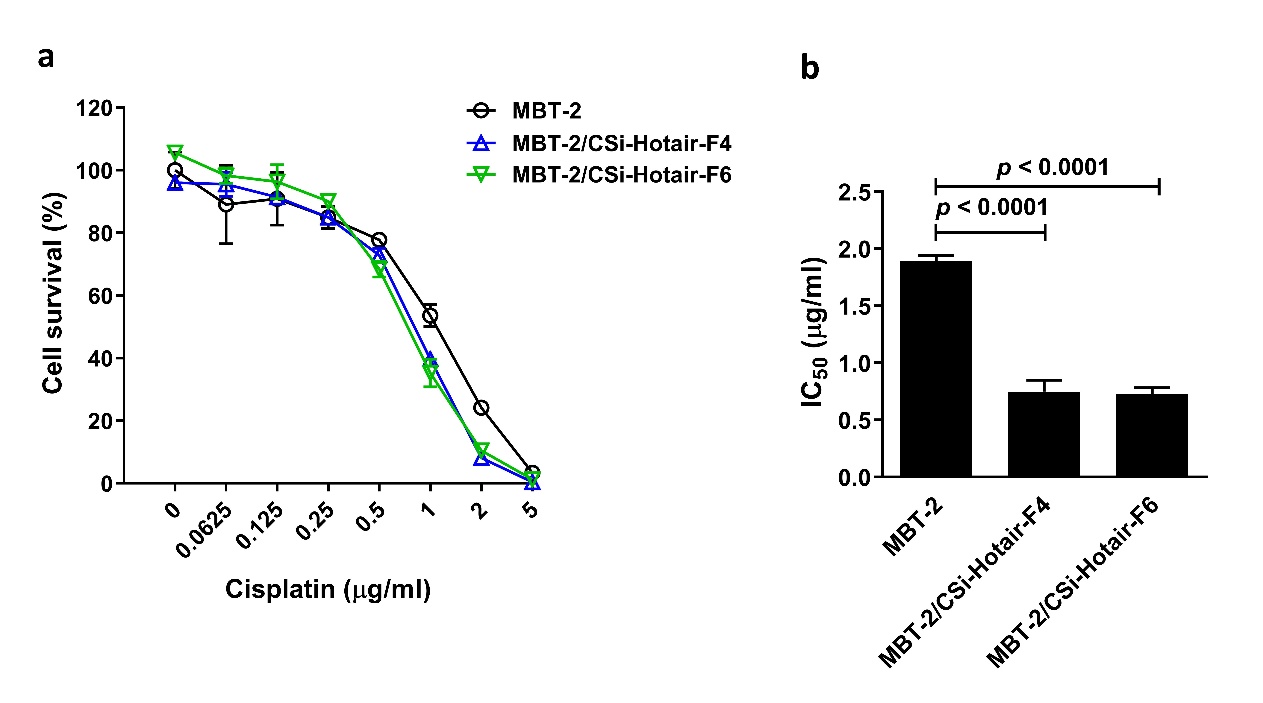
Fig. S4** Knockdown of Hotair enhances sensitivity to cisplatin in MBT-2 cells. Hotair-knockdown MBT-2 cells (MBT-2/CSi-Hotair-F4 and -F6) and parental cells (5 × 10^3^) that had been cultured in 96-well plates overnight were refed with the fresh medium containing various concentrations of cisplatin. After 24 h, cell viability was assessed with the colorimetric WST-8 assay (**a**), and IC_50_ values of cisplatin in different cells are determined (**b**). Values represent the relative cell survival, with the viability in the parental MBT-2 cells without cisplatin treatment arbitrarily set to 100. Values shown are mean ± SD (n = 3).
